# Supplementary material for: Changes in household food and drink purchases following restrictions on the advertisement of high fat, salt, and sugar products across the Transport for London network: A controlled interrupted time series analysis
Source: PLoS Med. 2022 Feb 17;19(2):e1003915. doi: 10.1371/journal.pmed.1003915 (PMC8853584; doi:10.1371/journal.pmed.1003915)
Supplement: S1 Table — (DOCX) [file pmed.1003915.s002.docx]

**S1 Table.** Definition of HFSS categories.

| **HFSS category** | **Included & excluded products** |
| --- | --- |
| Total HFSS | All food and drink products were included if classified as HFSS according to the NPM^1^ |
| Chocolate & confectionery* | Chocolate confectionery, sugar confectionery and sweet spreads (e.g. jams and chocolate spreads) |
| Puddings & biscuits | Biscuits, cakes, puddings, ice cream, custard, ready-to-eat icing, jellies and toaster pastries were included if classified as HFSS according to the NPM |
| Sugary drinks | Carbonated drinks, flavoured waters and milk-based drinks were included if classified as HFSS according to the NPM |
| Sugary cereals | Breakfast cereals were included if classified as HFSS according to the NPM |
| Savoury snacks | Crisps, popcorn, savoury crackers and biscuits, pork scratchings, poppadums and prawn crackers were included if classified as HFSS according to the NPM |
| HFSS, high in fat, sugar and salt. NPM, nutrient profiling model.  *All products in this food category were classified as HFSS  ^1^UK Department of Health. Nutrient Profiling Technical Guidance. London; 2011. | |
