# Supplementary material for: Changes in household food and drink purchases following restrictions on the advertisement of high fat, salt, and sugar products across the Transport for London network: A controlled interrupted time series analysis
Source: PLoS Med. 2022 Feb 17;19(2):e1003915. doi: 10.1371/journal.pmed.1003915 (PMC8853584; doi:10.1371/journal.pmed.1003915)
Supplement: S2 Table — (DOCX) [file pmed.1003915.s003.docx]

**S2 Table.** Coefficients for two-part model (energy).

|  | **Total HFSS** | | **Chocolate & Confectionery** | | **Puddings & Biscuits** | | **Sugary Drinks** | | **Sugary Cereals** | | **Savoury Snacks** | |
| --- | --- | --- | --- | --- | --- | --- | --- | --- | --- | --- | --- | --- |
| VARIABLES | Logit | Gamma | Logit | Gamma | Logit | Gamma | Logit | Gamma | Logit | Gamma | Logit | Gamma |
| **London* Intervention (level)** | 0.053 | 0.065 | 0.003 | 0.088 | -0.023 | 0.039 | 0.143 | 0.063 | 0.022 | 0.052 | 0.114 | -0.061 |
|  | (0.831) | (0.020) | (0.971) | (0.095) | (0.811) | (0.365) | (0.132) | (0.535) | (0.868) | (0.490) | (0.165) | (0.190) |
| **London* Intervention *Time (slope)** | -0.006 | -0.002 | -0.005 | -0.003 | -0.002 | -0.001 | -0.001 | 0.000 | 0.001 | -0.002 | -0.001 | 0.002 |
|  | (0.322) | (0.006) | (0.031) | (0.037) | (0.346) | (0.618) | (0.598) | (0.944) | (0.851) | (0.399) | (0.742) | (0.121) |
| Time | -0.005 | -0.006 | -0.007 | -0.007 | -0.008 | -0.005 | -0.007 | -0.002 | -0.000 | -0.000 | -0.004 | -0.004 |
|  | (0.266) | (<0.001) | (<0.001) | (<0.001) | (<0.001) | (<0.001) | (<0.001) | (0.225) | (0.824) | (0.724) | (0.006) | (<0.001) |
| London | -0.550 | -0.124 | -0.422 | -0.135 | -0.307 | -0.087 | -0.213 | 0.075 | -0.111 | 0.069 | -0.073 | 0.004 |
|  | (<0.001) | (<0.001) | (<0.001) | (<0.001) | (<0.001) | (0.002) | (0.001) | (0.214) | (0.135) | (0.095) | (0.183) | (0.887) |
| London*Time | 0.007 | 0.001 | 0.006 | 0.002 | 0.003 | -0.000 | -0.001 | -0.002 | -0.001 | 0.000 | -0.001 | -0.001 |
|  | (0.176) | (0.063) | (<0.001) | (0.076) | (0.132) | (0.597) | (0.769) | (0.422) | (0.609) | (0.931) | (0.723) | (0.333) |
| Intervention | -0.084 | -0.191 | 0.013 | -0.109 | -0.103 | -0.239 | -0.380 | -0.275 | 0.026 | -0.052 | -0.157 | -0.307 |
|  | (0.736) | (<0.001) | (0.866) | (0.016) | (0.235) | (<0.001) | (<0.001) | (0.003) | (0.803) | (0.410) | (0.026) | (<0.001) |
| Intervention *Time | 0.006 | 0.007 | 0.006 | 0.008 | 0.008 | 0.008 | 0.010 | 0.005 | -0.002 | 0.001 | 0.005 | 0.008 |
|  | (0.248) | (<0.001) | (<0.001) | (<0.001) | (<0.001) | (<0.001) | (<0.001) | (0.013) | (0.318) | (0.413) | (0.001) | (<0.001) |
| Weeks of Festival | -0.111 | 0.089 | 0.075 | 0.173 | -0.065 | 0.063 | 0.085 | 0.045 | -0.141 | 0.006 | 0.031 | 0.117 |
|  | (0.049) | (<0.001) | (<0.001) | (<0.001) | (0.001) | (<0.001) | (<0.001) | (0.015) | (<0.001) | (0.719) | (0.061) | (<0.001) |
| Number of Adults | 0.367 | 0.247 | 0.155 | 0.116 | 0.270 | 0.178 | 0.191 | 0.099 | 0.233 | 0.040 | 0.231 | 0.127 |
|  | (<0.001) | (<0.001) | (<0.001) | (<0.001) | (<0.001) | (<0.001) | (<0.001) | (<0.001) | (<0.001) | (0.003) | (<0.001) | (<0.001) |
| Number of Children | 0.342 | 0.199 | 0.152 | 0.093 | 0.359 | 0.158 | 0.076 | 0.031 | 0.301 | 0.037 | 0.228 | 0.111 |
|  | (<0.001) | (<0.001) | (<0.001) | (<0.001) | (<0.001) | (<0.001) | (0.008) | (0.249) | (<0.001) | (0.015) | (<0.001) | (<0.001) |
| Seasons (Winter=0) |  |  |  |  |  |  |  |  |  |  |  |  |
| Spring | 0.042 | -0.105 | -0.014 | -0.171 | -0.083 | -0.099 | -0.074 | -0.034 | 0.068 | 0.010 | -0.075 | -0.055 |
|  | (0.687) | (<0.001) | (0.667) | (<0.001) | (0.023) | (<0.001) | (0.032) | (0.370) | (0.128) | (0.726) | (0.018) | (0.003) |
| Summer | -0.059 | -0.160 | -0.194 | -0.277 | -0.086 | -0.127 | -0.123 | -0.040 | 0.087 | 0.001 | -0.111 | -0.121 |
|  | (0.434) | (<0.001) | (<0.001) | (<0.001) | (0.002) | (<0.001) | (<0.001) | (0.139) | (0.016) | (0.977) | (<0.001) | (<0.001) |
| Autumn | 0.025 | -0.083 | 0.126 | -0.011 | -0.011 | -0.071 | -0.114 | -0.047 | 0.009 | -0.016 | -0.080 | -0.117 |
|  | (0.643) | (<0.001) | (<0.001) | (0.373) | (0.609) | (<0.001) | (<0.001) | (0.020) | (0.743) | (0.323) | (<0.001) | (<0.001) |
| Sex of main shopper (Female=0) | | | | | | | | | | | | |
| Male | -0.190 | -0.057 | -0.237 | -0.026 | -0.205 | -0.036 | -0.099 | -0.015 | -0.257 | -0.020 | -0.003 | 0.049 |
|  |  | (0.009) | (<0.001) | (0.344) | (<0.001) | (0.181) | (0.077) | (0.762) | (<0.001) | (0.437) | (0.948) | (0.052) |
| Age of main shopper | 0.017 | 0.007 | 0.009 | 0.003 | 0.019 | 0.009 | 0.004 | 0.001 | 0.002 | 0.001 | -0.004 | 0.000 |
|  |  | (<0.001) | (<0.001) | (<0.001) | (<0.001) | (<0.001) | (0.032) | (0.628) | (0.385) | (0.279) | (0.025) | (0.791) |
| Socioeconomic position (High SEP=0) | | | | | | | | | | | | |
| Middle SEP | 0.215 | 0.118 | 0.191 | 0.016 | 0.221 | 0.088 | 0.142 | 0.027 | 0.060 | -0.064 | 0.152 | 0.072 |
|  | (0.033) | (<0.001) | (<0.001) | (0.565) | (<0.001) | (0.001) | (0.019) | (0.623) | (0.403) | (0.035) | (0.003) | (0.006) |
| Low SEP | 0.213 | 0.158 | 0.285 | 0.085 | 0.210 | 0.159 | 0.412 | 0.204 | -0.129 | -0.059 | 0.086 | 0.130 |
|  | (<0.001) | (<0.001) | (<0.001) | (0.022) | (0.004) | (<0.001) | (<0.001) | (0.004) | (0.168) | (0.132) | (0.227) | (0.001) |
| Constant | 2.066 | 8.743 | -0.597 | 7.660 | -0.538 | 7.521 | -1.504 | 6.513 | -2.451 | 7.916 | -0.300 | 7.362 |
|  | (<0.001) | (<0.001) | (<0.001) | (<0.001) | (<0.001) | (<0.001) | (<0.001) | (<0.001) | (<0.001) | (<0.001) | (0.023) | (<0.001) |
| Observations | 139,193 | 139,193 | 139,193 | 139,193 | 139,193 | 139,193 | 139,193 | 139,193 | 139,193 | 139,193 | 139,193 | 139,193 |

SEP, socioeconomic position. London*Intervention=post-intervention period in London (level), London*Intervention*Time=post-intervention trend in London (slope), London*Time=trend in London, Intervention*Time=post-intervention trend in the North of England. P-values in parentheses.
