# Supplementary material for: Changes in household food and drink purchases following restrictions on the advertisement of high fat, salt, and sugar products across the Transport for London network: A controlled interrupted time series analysis
Source: PLoS Med. 2022 Feb 17;19(2):e1003915. doi: 10.1371/journal.pmed.1003915 (PMC8853584; doi:10.1371/journal.pmed.1003915)
Supplement: S3 Table — (DOCX) [file pmed.1003915.s004.docx]

**S3 Table.** Coefficients for two-part model (fat).

|  | **Total HFSS** | | **Chocolate & Confectionery** | | **Puddings & Biscuits** | | **Sugary Drinks** | | **Sugary Cereals** | | **Savoury Snacks** | |
| --- | --- | --- | --- | --- | --- | --- | --- | --- | --- | --- | --- | --- |
| VARIABLES | Logit | Gamma | Logit | Gamma | Logit | Gamma | Logit | Gamma | Logit | Gamma | Logit | Gamma |
| **London* Intervention (level)** | 0.033 | 0.056 | 0.006 | 0.146 | -0.023 | 0.027 | 0.067 | 0.175 | 0.022 | -0.010 | 0.114 | -0.074 |
|  | (0.892) | (0.082) | (0.944) | (0.019) | (0.807) | (0.540) | (0.535) | (0.193) | (0.866) | (0.942) | (0.165) | (0.150) |
| **London* Intervention *Time (slope)** | -0.005 | -0.002 | -0.004 | -0.004 | -0.002 | -0.000 | -0.002 | -0.004 | 0.001 | -0.002 | -0.001 | 0.002 |
|  | (0.399) | (0.012) | (0.033) | (0.011) | (0.324) | (0.816) | (0.539) | (0.236) | (0.853) | (0.632) | (0.742) | (0.080) |
| Time | -0.004 | -0.005 | -0.007 | -0.008 | -0.008 | -0.005 | -0.002 | 0.003 | -0.000 | 0.004 | -0.004 | -0.004 |
|  | (0.328) | (<0.001) | (<0.001) | (<0.001) | (<0.001) | (<0.001) | (0.232) | (0.102) | (0.826) | (0.040) | (0.006) | (<0.001) |
| London | -0.540 | -0.087 | -0.433 | -0.079 | -0.310 | -0.069 | -0.369 | -0.123 | -0.111 | 0.166 | -0.073 | 0.017 |
|  | (<0.001) | (<0.001) | (<0.001) | (0.045) | (<0.001) | (0.018) | (<0.001) | (0.137) | (0.135) | (0.024) | (0.183) | (0.588) |
| London*Time | 0.006 | 0.001 | 0.006 | 0.003 | 0.003 | -0.001 | 0.000 | 0.003 | -0.001 | 0.000 | -0.001 | -0.001 |
|  | (0.218) | (0.051) | (<0.001) | (0.063) | (0.104) | (0.502) | (0.880) | (0.342) | (0.609) | (0.930) | (0.723) | (0.151) |
| Intervention | 0.117 | -0.150 | 0.007 | -0.112 | -0.107 | -0.212 | -0.051 | -0.003 | 0.026 | 0.050 | -0.157 | -0.329 |
|  | (0.631) | (<0.001) | (0.926) | (0.045) | (0.226) | (<0.001) | (0.577) | (0.984) | (0.808) | (0.642) | (0.026) | (<0.001) |
| Intervention *Time | 0.002 | 0.007 | 0.006 | 0.008 | 0.009 | 0.008 | 0.001 | -0.001 | -0.002 | -0.002 | 0.005 | 0.009 |
|  | (0.653) | (<0.001) | (<0.001) | (<0.001) | (<0.001) | (<0.001) | (0.609) | (0.605) | (0.319) | (0.339) | (0.001) | (<0.001) |
| Weeks of Festival | -0.122 | 0.099 | 0.087 | 0.183 | -0.063 | 0.067 | -0.007 | 0.012 | -0.141 | 0.024 | 0.031 | 0.126 |
|  | (0.025) | (<0.001) | (<0.001) | (<0.001) | (0.001) | (<0.001) | (0.745) | (0.663) | (<0.001) | (0.303) | (0.061) | (<0.001) |
| Number of Adults | 0.361 | 0.256 | 0.151 | 0.122 | 0.272 | 0.178 | 0.195 | 0.107 | 0.233 | 0.043 | 0.231 | 0.127 |
|  | (<0.001) | (<0.001) | (<0.001) | (<0.001) | (<0.001) | (<0.001) | (<0.001) | (0.004) | (<0.001) | (0.067) | (<0.001) | (<0.001) |
| Number of Children | 0.350 | 0.196 | 0.149 | 0.092 | 0.360 | 0.147 | 0.118 | 0.035 | 0.301 | 0.039 | 0.228 | 0.101 |
|  | (<0.001) | (<0.001) | (<0.001) | (<0.001) | (<0.001) | (<0.001) | (<0.001) | (0.289) | (<0.001) | (0.136) | (<0.001) | (<0.001) |
| Seasons (Winter=0) |  |  |  |  |  |  |  |  |  |  |  |  |
| Spring | -0.020 | -0.108 | -0.030 | -0.190 | -0.084 | -0.089 | -0.070 | -0.093 | 0.068 | 0.081 | -0.075 | -0.044 |
|  | (0.845) | (<0.001) | (0.350) | (<0.001) | (0.021) | (<0.001) | (0.069) | (0.131) | (0.127) | (0.086) | (0.018) | (0.026) |
| Summer | -0.091 | -0.163 | -0.216 | -0.339 | -0.091 | -0.103 | -0.170 | -0.061 | 0.087 | 0.083 | -0.111 | -0.125 |
|  | (0.214) | (<0.001) | (<0.001) | (<0.001) | (0.001) | (<0.001) | (<0.001) | (0.137) | (0.016) | (0.038) | (<0.001) | (<0.001) |
| Autumn | 0.018 | -0.093 | 0.118 | -0.049 | -0.009 | -0.072 | -0.025 | 0.012 | 0.008 | 0.037 | -0.080 | -0.124 |
|  | (0.727) | (<0.001) | (<0.001) | (0.001) | (0.671) | (<0.001) | (0.272) | (0.692) | (0.746) | (0.212) | (<0.001) | (<0.001) |
| Sex of main shopper (Female=0) | | | | | | | | | | | | |
| Male | -0.209 | -0.031 | -0.236 | -0.022 | -0.199 | -0.018 | -0.117 | -0.041 | -0.257 | 0.039 | -0.003 | 0.070 |
|  | (0.014) | (0.188) | (<0.001) | (0.473) | (<0.001) | (0.517) | (0.051) | (0.566) | (<0.001) | (0.470) | (0.948) | (0.010) |
| Age of main shopper | 0.017 | 0.007 | 0.008 | 0.000 | 0.019 | 0.008 | 0.012 | 0.009 | 0.002 | 0.001 | -0.004 | 0.001 |
|  | (<0.001) | (<0.001) | (<0.001) | (0.631) | (<0.001) | (<0.001) | (0.057) | (<0.001) | (0.386) | (0.531) | (0.025) | (0.411) |
| Socioeconomic position (High SEP=0) | | | | | | | | | | | | |
| Middle SEP | 0.204 | 0.100 | 0.196 | -0.033 | 0.223 | 0.080 | 0.125 | -0.033 | 0.059 | -0.189 | 0.152 | 0.057 |
|  | (0.019) | (<0.001) | (<0.001) | (0.299) | (<0.001) | (0.007) | (0.057) | (0.646) | (0.404) | (<0.001) | (0.003) | (0.041) |
| Low SEP | 0.197 | 0.142 | 0.286 | 0.023 | 0.212 | 0.159 | 0.345 | 0.078 | -0.129 | -0.152 | 0.086 | 0.114 |
|  | (0.120) | (<0.001) | (<0.001) | (0.572) | (0.004) | (<0.001) | (<0.001) | (0.414) | (0.168) | (0.040) | (0.227) | (0.006) |
| Constant | 1.966 | 5.878 | -0.600 | 4.737 | -0.551 | 4.394 | -2.346 | 2.010 | -2.451 | 3.840 | -0.300 | 4.491 |
|  | (<0.001) | (<0.001) | (<0.001) | (<0.001) | (<0.001) | (<0.001) | (<0.001) | (<0.001) | (<0.001) | (<0.001) | (0.023) | (<0.001) |
| Observations | 139,193 | 139,193 | 139,193 | 139,193 | 139,193 | 139,193 | 139,193 | 139,193 | 139,193 | 139,193 | 139,193 | 139,193 |

SEP, socioeconomic position. London*Intervention=post-intervention period in London (level), London*Intervention*Time=post-intervention trend in London (slope), London*Time=trend in London, Intervention*Time=post-intervention trend in the North of England. P-values in parentheses.
