# Supplementary material for: Changes in household food and drink purchases following restrictions on the advertisement of high fat, salt, and sugar products across the Transport for London network: A controlled interrupted time series analysis
Source: PLoS Med. 2022 Feb 17;19(2):e1003915. doi: 10.1371/journal.pmed.1003915 (PMC8853584; doi:10.1371/journal.pmed.1003915)
Supplement: S4 Table — (DOCX) [file pmed.1003915.s005.docx]

**S4 Table.** Coefficients for two-part model (saturated fat).

|  | **Total HFSS** | | **Chocolate & Confectionery** | | **Puddings & Biscuits** | | **Sugary Drinks** | | **Sugary Cereals** | | **Savoury Snacks** | |
| --- | --- | --- | --- | --- | --- | --- | --- | --- | --- | --- | --- | --- |
| VARIABLES | Logit | Gamma | Logit | Gamma | Logit | Gamma | Logit | Gamma | Logit | Gamma | Logit | Gamma |
| **London* Intervention (level)** | -0.010 | 0.043 | 0.001 | 0.143 | -0.023 | -0.009 | 0.049 | 0.141 | 0.024 | 0.007 | 0.113 | -0.023 |
|  | (0.967) | (0.155) | (0.991) | (0.021) | (0.811) | (0.848) | (0.650) | (0.257) | (0.854) | (0.966) | (0.168) | (0.767) |
| **London* Intervention *Time (slope)** | -0.004 | -0.002 | -0.005 | -0.005 | -0.002 | 0.001 | -0.001 | -0.004 | 0.001 | -0.003 | -0.001 | 0.002 |
|  | (0.481) | (0.027) | (0.031) | (0.004) | (0.334) | (0.447) | (0.721) | (0.228) | (0.873) | (0.449) | (0.776) | (0.408) |
| Time | -0.004 | -0.006 | -0.008 | -0.008 | -0.008 | -0.005 | -0.001 | 0.004 | -0.000 | 0.002 | -0.004 | -0.005 |
|  | (0.338) | (<0.001) | (<0.001) | (<0.001) | (<0.001) | (<0.001) | (0.466) | (0.040) | (0.841) | (0.401) | (0.008) | (<0.001) |
| London | -0.528 | -0.137 | -0.442 | -0.177 | -0.311 | -0.061 | -0.336 | -0.173 | -0.113 | 0.123 | -0.072 | 0.037 |
|  | (<0.001) | (<0.001) | (<0.001) | (<0.001) | (<0.001) | (0.047) | (<0.001) | (0.042) | (0.129) | (0.174) | (0.193) | (0.344) |
| London*Time | 0.005 | 0.001 | 0.006 | 0.004 | 0.003 | -0.002 | -0.001 | 0.004 | -0.001 | 0.001 | -0.001 | -0.001 |
|  | (0.241) | (0.030) | (<0.001) | (0.006) | (0.104) | (0.119) | (0.764) | (0.153) | (0.623) | (0.735) | (0.674) | (0.486) |
| Intervention | 0.160 | -0.165 | -0.007 | -0.110 | -0.113 | -0.195 | -0.026 | 0.034 | 0.030 | 0.012 | -0.154 | -0.311 |
|  | (0.506) | (<0.001) | (0.921) | (0.052) | (0.196) | (<0.001) | (0.777) | (0.807) | (0.774) | (0.923) | (0.028) | (<0.001) |
| Intervention *Time | 0.002 | 0.007 | 0.007 | 0.009 | 0.009 | 0.007 | 0.000 | -0.003 | -0.003 | -0.000 | 0.005 | 0.009 |
|  | (0.760) | (<0.001) | (<0.001) | (<0.001) | (<0.001) | (<0.001) | (0.832) | (0.236) | (0.295) | (0.870) | (0.001) | (<0.001) |
| Weeks of Festival | -0.131 | 0.114 | 0.097 | 0.214 | -0.061 | 0.069 | -0.014 | 0.014 | -0.141 | -0.027 | 0.032 | 0.143 |
|  | (0.014) | (<0.001) | (<0.001) | (<0.001) | (0.001) | (<0.001) | (0.512) | (0.581) | (<0.001) | (0.327) | (0.060) | (<0.001) |
| Number of Adults | 0.364 | 0.234 | 0.150 | 0.123 | 0.272 | 0.177 | 0.200 | 0.097 | 0.233 | 0.031 | 0.232 | 0.103 |
|  | (<0.001) | (<0.001) | (<0.001) | (<0.001) | (<0.001) | (<0.001) | (<0.001) | (0.01) | (<0.001) | (0.262) | (<0.001) | (<0.001) |
| Number of Children | 0.348 | 0.183 | 0.148 | 0.075 | 0.360 | 0.146 | 0.114 | 0.021 | 0.300 | 0.046 | 0.228 | 0.076 |
|  | (<0.001) | (<0.001) | (<0.001) | (<0.001) | (<0.001) | (<0.001) | (<0.001) | (0.505) | (<0.001) | (0.115) | (<0.001) | (<0.001) |
| Seasons (Winter=0) | | | | | | | | | | | | |
| Spring | -0.039 | -0.110 | -0.028 | -0.206 | -0.085 | -0.074 | -0.079 | -0.070 | 0.069 | 0.059 | -0.075 | -0.079 |
|  | (0.697) | (<0.001) | (0.383) | (<0.001) | (0.019) | (<0.001) | (0.042) | (0.250) | (0.122) | (0.268) | (0.018) | (0.011) |
| Summer | -0.096 | -0.163 | -0.224 | -0.392 | -0.093 | -0.068 | -0.171 | -0.055 | 0.088 | 0.027 | -0.111 | -0.176 |
|  | (0.185) | (<0.001) | (<0.001) | (<0.001) | (0.001) | (<0.001) | (<0.001) | (0.157) | (0.015) | (0.533) | (<0.001) | (<0.001) |
| Autumn | 0.018 | -0.097 | 0.119 | -0.054 | -0.011 | -0.078 | -0.025 | 0.017 | 0.010 | -0.001 | -0.080 | -0.150 |
|  | (0.735) | (<0.001) | (<0.001) | (<0.001) | (0.607) | (<0.001) | (0.268) | (0.548) | (0.703) | (0.985) | (<0.001) | (<0.001) |
| Sex of main shopper (Female=0) | | | | | | | | | | | | |
| Male | -0.200 | -0.059 | -0.228 | -0.043 | -0.198 | -0.009 | -0.118 | -0.039 | -0.257 | 0.061 | -0.003 | 0.072 |
|  | (0.018) | (0.016) | (<0.001) | (0.184) | (<0.001) | (0.753) | (0.053) | (0.587) | (<0.001) | (0.345) | (0.958) | (0.044) |
| Age of main shopper | 0.017 | 0.008 | 0.007 | 0.003 | 0.019 | 0.008 | 0.013 | 0.010 | 0.002 | 0.003 | -0.004 | 0.004 |
|  | (<0.001) | (<0.001) | (<0.001) | (0.007) | (<0.001) | (<0.001) | (<0.001) | (<0.001) | (0.380) | (0.138) | (0.025) | (0.002) |
| Socioeconomic position (High SEP=0) | | | | | | | | | | | | |
| Middle SEP | -0.214 | 0.073 | 0.194 | -0.028 | 0.226 | 0.073 | 0.121 | -0.006 | 0.058 | -0.197 | 0.153 | 0.024 |
|  | (0.014) | (0.004) | (<0.001) | (0.406) | (<0.001) | (0.020) | (0.069) | (0.938) | (0.415) | (0.001) | (0.003) | (0.489) |
| Low SEP | 0.202 | 0.077 | 0.283 | 0.048 | 0.217 | 0.142 | 0.333 | 0.117 | -0.131 | -0.123 | 0.086 | 0.043 |
|  | (0.109) | (0.018) | (<0.001) | (0.277) | (0.003) | (0.001) | (<0.001) | (0.240) | (0.162) | (0.172) | (0.226) | (0.365) |
| Constant | 1.959 | 5.036 | -0.583 | 4.025 | -0.554 | 3.750 | -2.446 | 1.714 | -2.453 | 2.649 | -0.303 | 2.563 |
|  | (<0.001) | (<0.001) | (<0.001) | (<0.001) | (<0.001) | (<0.001) | (<0.001) | (<0.001) | (<0.001) | (<0.001) | (0.022) | (<0.001) |
| Observations | 139,193 | 139,193 | 139,193 | 139,193 | 139,193 | 139,193 | 139,193 | 139,193 | 139,193 | 139,193 | 139,193 | 139,193 |

SEP, socioeconomic position. London*Intervention=post-intervention period in London (level), London*Intervention*Time=post-intervention trend in London (slope), London*Time=trend in London, Intervention*Time=post-intervention trend in the North of England. P-values in parentheses.
