# Supplementary material for: Changes in household food and drink purchases following restrictions on the advertisement of high fat, salt, and sugar products across the Transport for London network: A controlled interrupted time series analysis
Source: PLoS Med. 2022 Feb 17;19(2):e1003915. doi: 10.1371/journal.pmed.1003915 (PMC8853584; doi:10.1371/journal.pmed.1003915)
Supplement: S5 Table — (DOCX) [file pmed.1003915.s006.docx]

**S5 Table.** Coefficients for two-part model (sugar).

|  |  | **Total HFSS** | | **Chocolate & Confectionery** | | **Puddings & Biscuits** | | **Sugary Drinks** | | **Sugary Cereals** | | **Savoury Snacks** | |
| --- | --- | --- | --- | --- | --- | --- | --- | --- | --- | --- | --- | --- | --- |
|  | VARIABLES | Logit | Gamma | Logit | Gamma | Logit | Gamma | Logit | Gamma | Logit | Gamma | Logit | Gamma |
| **London* Intervention (level)** | | 0.070 | 0.103 | -0.008 | 0.062 | -0.025 | 0.049 | 0.139 | 0.091 | 0.022 | 0.093 | 0.104 | -0.022 |
|  |  | (0.769) | (0.010) | (0.919) | (0.264) | (0.797) | (0.288) | (0.141) | (0.404) | (0.868) | (0.254) | (0.206) | (0.811) |
| **London* Intervention *Time (slope)** | | -0.007 | -0.003 | -0.004 | -0.003 | -0.002 | 0.000 | -0.001 | 0.000 | 0.001 | -0.002 | -0.000 | 0.001 |
|  |  | (0.237) | (0.004) | (0.040) | (0.085) | (0.369) | (0.749) | (0.610) | (0.940) | (0.851) | (0.324) | (0.828) | (0.620) |
| Time | | -0.005 | -0.008 | -0.006 | -0.007 | -0.008 | -0.006 | -0.007 | -0.005 | -0.000 | -0.001 | -0.004 | -0.004 |
|  | | (0.200) | (<0.001) | (<0.001) | (<0.001) | (<0.001) | (<0.001) | (<0.001) | (0.012) | (0.824) | (0.293) | (0.007) | (0.007) |
| London | | -0.553 | -0.200 | -0.418 | -0.189 | -0.305 | -0.080 | -0.213 | 0.169 | -0.111 | -0.004 | -0.102 | 0.056 |
|  | | (<0.001) | (<0.001) | (<0.001) | (<0.001) | (<0.001) | (0.005) | (0.001) | (0.013) | (0.135) | (0.929) | (0.065) | (0.216) |
| London*Time | | 0.007 | 0.002 | 0.006 | 0.002 | 0.003 | -0.002 | -0.001 | -0.002 | -0.001 | 0.001 | -0.000 | -0.000 |
|  | | (0.128) | (0.054) | (0.001) | (0.102) | (0.145) | (0.033) | (0.765) | (0.380) | (0.609) | (0.718) | (0.807) | (0.828) |
| Intervention | | -0.143 | -0.268 | 0.008 | -0.097 | -0.101 | -0.306 | -0.387 | -0.391 | 0.026 | -0.032 | -0.156 | -0.241 |
|  | | (0.534) | (<0.001) | (0.910) | (0.034) | (0.254) | (<0.001) | (<0.001) | (<0.001) | (0.803) | (0.633) | (0.026) | (0.001) |
| Intervention *Time | | 0.007 | 0.010 | 0.006 | 0.008 | 0.008 | 0.009 | 0.010 | 0.008 | -0.002 | 0.001 | 0.005 | 0.007 |
|  | | (0.146) | (<0.001) | (<0.001) | (<0.001) | (<0.001) | (<0.001) | (<0.001) | (<0.001) | (0.318) | (0.495) | (0.001) | (<0.001) |
| Weeks of Festival | | -0.103 | 0.094 | 0.077 | 0.178 | -0.063 | 0.074 | 0.086 | 0.091 | -0.141 | 0.009 | 0.034 | 0.121 |
|  | | (0.050) | (<0.001) | (<0.001) | (<0.001) | (0.001) | (<0.001) | (<0.001) | (<0.001) | (<0.001) | 0.577) | (0.041) | (<0.001) |
| Number of Adults | | 0.395 | 0.236 | 0.155 | 0.112 | 0.270 | 0.167 | 0.192 | 0.107 | 0.233 | 0.041 | 0.238 | 0.098 |
|  | | (<0.001) | (<0.001) | (<0.001) | (<0.001) | (<0.001) | (<0.001) | (<0.001) | (0.001) | (<0.001) | (0.010) | (<0.001) | (<0.001) |
| Number of Children | | 0.348 | 0.201 | 0.152 | 0.097 | 0.361 | 0.168 | 0.076 | 0.016 | 0.301 | 0.044 | 0.227 | 0.123 |
|  | | (<0.001) | (<0.001) | (<0.001) | (<0.001) | (<0.001) | (<0.001) | (0.009) | (0.653) | (<0.001) | (0.006) | (<0.001) | (<0.001) |
| Seasons (Winter=0) | | | | | | | | | | | | | |
| Spring | | 0.076 | -0.108 | -0.012 | -0.174 | -0.084 | -0.101 | -0.072 | -0.022 | 0.068 | -0.018 | -0.076 | -0.062 |
|  | | (0.422) | (<0.001) | (0.707) | (<0.001) | (0.021) | (<0.001) | (0.038) | (0.546) | (0.128) | (0.543) | (0.016) | (0.049) |
| Summer | | -0.047 | -0.179 | -0.194 | -0.261 | -0.088 | -0.122 | -0.124 | -0.020 | 0.087 | -0.019 | -0.115 | -0.133 |
|  | | (0.499) | (<0.001) | (<0.001) | (<0.001) | (0.002) | (<0.001) | (<0.001) | (0.486) | (0.016) | (0.440) | (<0.001) | (<0.001) |
| Autumn | | 0.035 | -0.052 | 0.127 | 0.008 | -0.012 | -0.079 | -0.115 | -0.077 | 0.009 | -0.026 | -0.080 | -0.109 |
|  | | (0.492) | (<0.001) | (<0.001) | (0.541) | (0.549) | (<0.001) | (<0.001) | (<0.001) | (0.743) | (0.117) | (<0.001) | (<0.001) |
| Sex of main shopper (Female=0) | | | | | | | | | | | | | |
| Male | | -0.170 | -0.126 | -0.231 | -0.038 | -0.204 | -0.062 | -0.098 | 0.002 | -0.257 | -0.013 | -0.010 | 0.001 |
|  | | (0.052) | (<0.001) | (<0.001) | (0.200) | (<0.001) | (0.022) | (0.079) | (0.974) | (<0.001) | (0.693) | (0.850) | (0.984) |
| Age of main shopper | | 0.017 | 0.009 | 0.009 | 0.006 | 0.019 | 0.009 | 0.005 | -0.006 | 0.002 | 0.001 | -0.004 | -0.002 |
|  | | (<0.001) | (<0.001) | (<0.001) | (<0.001) | (<0.001) | (<0.001) | (0.028) | (0.013) | (0.385) | (0.450) | (0.019) | (0.168) |
| Socioeconomic position (High SEP=0) | | | | | | | | | | | | | |
| Middle SEP | | 0.213 | 0.130 | 0.189 | 0.040 | 0.220 | 0.068 | 0.144 | 0.047 | 0.060 | -0.032 | 0.165 | 0.098 |
|  | | (0.016) | (<0.001) | (<0.001) | (0.171) | (<0.001) | (0.019) | (0.018) | (0.486) | (0.403) | (0.364) | (0.001) | (0.012) |
| Low SEP | | 0.227 | 0.205 | 0.290 | 0.114 | 0.211 | 0.113 | 0.416 | 0.249 | -0.129 | -0.001 | 0.095 | 0.102 |
|  | | (0.070) | (<0.001) | (<0.001) | (0.004) | (0.004) | (0.004) | (<0.001) | (0.004) | (0.168) | (0.981) | (0.182) | (0.047) |
| Constant | | 1.863 | 5.787 | -0.603 | 5.387 | -0.540 | 5.074 | -1.515 | 5.104 | -2.451 | 5.186 | -0.335 | 2.776 |
|  | | (<0.001) | (<0.001) | (<0.001) | (<0.001) | (<0.001) | (<0.001) | (<0.001) | (<0.001) | (<0.001) | (<0.001) | (0.012) | (<0.001) |
| Observations | | 139,193 | 139,193 | 139,193 | 139,193 | 139,193 | 139,193 | 139,193 | 139,193 | 139,193 | 139,193 | 139,193 | 139,193 |

SEP, socioeconomic position. London*Intervention=post-intervention period in London (level), London*Intervention*Time=post-intervention trend in London (slope), London*Time=trend in London, Intervention*Time=post-intervention trend in the North of England. P-values in parentheses.
