# Supplementary material for: Changes in household food and drink purchases following restrictions on the advertisement of high fat, salt, and sugar products across the Transport for London network: A controlled interrupted time series analysis
Source: PLoS Med. 2022 Feb 17;19(2):e1003915. doi: 10.1371/journal.pmed.1003915 (PMC8853584; doi:10.1371/journal.pmed.1003915)
Supplement: S6 Table — (DOCX) [file pmed.1003915.s007.docx]

**S6 Table.** Coefficients for two-part model (salt).

|  |  | **Total HFSS** | | **Chocolate & Confectionery** | | **Puddings & Biscuits** | | **Sugary Drinks** | | **Sugary Cereals** | | **Savoury Snacks** | |
| --- | --- | --- | --- | --- | --- | --- | --- | --- | --- | --- | --- | --- | --- |
|  | VARIABLES | Logit | Gamma | Logit | Gamma | Logit | Gamma | Logit | Gamma | Logit | Gamma | Logit | Gamma |
| **London* Intervention (level)** | | 0.040 | 0.033 | 0.018 | 0.148 | -0.020 | 0.030 | 0.141 | 0.282 | 0.015 | 0.012 | 0.109 | -0.072 |
|  |  | (0.870) | (0.620) | (0.828) | (0.041) | (0.831) | (0.620) | (0.172) | (0.111) | (0.910) | (0.912) | (0.184) | (0.192) |
| **London* Intervention *Time (slope)** | | -0.006 | -0.001 | -0.005 | -0.003 | -0.003 | -0.002 | -0.002 | -0.007 | 0.001 | 0.001 | -0.001 | 0.003 |
|  |  | (0.331) | (0.554) | (0.025) | (0.095) | (0.297) | (0.206) | (0.422) | (0.109) | (0.828) | (0.848) | (0.795) | (0.028) |
| Time | | -0.005 | -0.002 | -0.007 | -0.005 | -0.008 | -0.005 | -0.004 | 0.002 | -0.001 | -0.005 | -0.004 | -0.004 |
|  | | (0.279) | (0.051) | (<0.001) | (<0.001) | (<0.001) | (<0.001) | (0.013) | (0.450) | (0.807) | (0.007) | (0.008) | (<0.001) |
| London | | -0.535 | -0.054 | -0.455 | -0.125 | -0.325 | -0.112 | -0.314 | -0.163 | -0.110 | 0.103 | -0.069 | 0.085 |
|  | | (<0.001) | (0.195) | (<0.001) | (0.005) | (<0.001) | (0.002) | (<0.001) | (0.099) | (0.138) | (0.061) | (0.212) | (0.010) |
| London*Time | | 0.006 | 0.001 | 0.006 | 0.001 | 0.003 | 0.002 | -0.000 | 0.004 | -0.001 | -0.000 | -0.001 | -0.002 |
|  | | (0.209) | (0.543) | (<0.001) | (0.382) | (0.086) | (0.092) | (0.892) | (0.267) | (0.615) | (0.826) | (0.635) | (0.024) |
| Intervention | | -0.061 | -0.135 | 0.023 | -0.129 | -0.092 | -0.110 | -0.209 | -0.159 | 0.032 | -0.051 | -0.153 | -0.264 |
|  | | (0.800) | (0.024) | (0.757) | (0.038) | (0.295) | (0.027) | (0.017) | (0.354) | (0.761) | (0.572) | (0.029) | (<0.001) |
| Intervention *Time | | 0.006 | 0.004 | 0.006 | 0.007 | 0.008 | 0.006 | 0.005 | 0.001 | -0.003 | 0.003 | 0.005 | 0.007 |
|  | | (0.250) | (0.005) | (0.001) | (<0.001) | (<0.001) | (<0.001) | (0.017) | (0.666) | (0.307) | (0.190) | (0.001) | (<0.001) |
| Weeks of Festival | | -0.140 | 0.041 | 0.079 | 0.133 | -0.061 | 0.034 | 0.014 | -0.010 | -0.139 | 0.027 | 0.032 | 0.117 |
|  | | (0.010) | (0.018) | (<0.001) | (<0.001) | (0.002) | (0.014) | (0.491) | 0.744) | (<0.001) | (0.209) | (0.054) | (<0.001) |
| Number of Adults | | 0.372 | 0.267 | 0.150 | 0.119 | 0.271 | 0.179 | 0.187 | 0.083 | 0.233 | 0.075 | 0.231 | 0.118 |
|  | | (<0.001) | (<0.001) | (<0.001) | (<0.001) | (<0.001) | (<0.001) | (<0.001) | (0.010) | (<0.001) | (0.001) | (<0.001) | (<0.001) |
| Number of Children | | 0.334 | 0.166 | 0.155 | 0.077 | 0.361 | 0.145 | 0.097 | 0.085 | 0.301 | 0.051 | 0.228 | 0.114 |
|  | | (<0.001) | (<0.001) | (<0.001) | (<0.001) | (<0.001) | (<0.001) | (0.001) | (0.011) | (<0.001) | (0.021) | (<0.001) | (<0.001) |
| Seasons (Winter=0) | | | | | | | | | | | | | |
| Spring | | 0.008 | -0.060 | -0.027 | -0.161 | -0.078 | -0.087 | -0.046 | -0.070 | 0.067 | -0.103 | -0.075 | -0.053 |
|  | | (0.939) | (0.056) | (0.403) | (<0.001) | (0.032) | (<0.001) | (0.217) | (0.303) | (0.132) | (0.008) | (0.017) | (0.008) |
| Summer | | -0.084 | -0.081 | -0.209 | -0.211 | -0.083 | -0.124 | -0.123 | -0.148 | 0.089 | -0.073 | -0.112 | -0.106 |
|  | | (0.253) | (0.001) | (<0.001) | (<0.001) | (0.003) | (<0.001) | (<0.001) | (0.002) | (0.015) | (0.025) | (<0.001) | (<0.001) |
| Autumn | | 0.011 | -0.053 | 0.125 | 0.035 | -0.005 | -0.043 | -0.054 | 0.014 | 0.009 | -0.064 | -0.081 | -0.114 |
|  | | (0.830) | (0.003) | (<0.001) | (0.037) | (0.821) | (0.003) | (0.013) | (0.688) | (0.740) | (0.005) | (<0.001) | (<0.001) |
| Sex of main shopper (Female=0) | | | | | | | | | | | | | |
| Male | | -0.185 | -0.007 | -0.223 | -0.024 | -0.201 | -0.073 | -0.105 | -0.015 | -0.256 | -0.130 | -0.003 | 0.063 |
|  | | (0.031) | (0.794) | (<0.001) | (0.451) | (<0.001) | (0.011) | (0.073) | (0.827) | (<0.001) | (0.007) | (0.959) | (0.019) |
| Age of main shopper | | 0.017 | 0.005 | 0.008 | 0.003 | 0.019 | 0.007 | 0.009 | 0.012 | 0.002 | -0.003 | -0.004 | -0.001 |
|  | | (<0.001) | (<0.001) | (<0.001) | (0.013) | (<0.001) | (<0.001) | (<0.001) | (<0.001) | (0.403) | (0.148) | (0.026) | (0.445) |
| Socioeconomic position (High SEP=0) | | | | | | | | | | | | | |
| Middle SEP | | 0.197 | 0.124 | 0.195 | 0.034 | 0.229 | 0.101 | 0.156 | -0.012 | 0.058 | -0.022 | 0.150 | 0.038 |
|  | | (0.025) | (<0.001) | (<0.001) | (0.334) | (<0.001) | (0.001) | (0.015) | (0.867) | (0.414) | (0.664) | (0.003) | (0.159) |
| Low SEP | | 0.213 | 0.165 | 0.283 | 0.113 | 0.225 | 0.163 | 0.415 | 0.070 | -0.130 | 0.136 | 0.086 | 0.093 |
|  | | (0.097) | (<0.001) | (<0.001) | (0.019) | (0.002) | (<0.001) | (<0.001) | (0.439) | (0.166) | (0.031) | (0.226) | (0.020) |
| Constant | | 2.011 | 3.181 | -0.605 | 0.159 | -0.585 | 0.813 | -2.030 | -0.414 | -2.450 | 1.488 | -0.303 | 1.720 |
|  | | (<0.001) | (<0.001) | (<0.001) | (0.087) | (<0.001) | (<0.001) | (<0.001) | (0.014) | (<0.001) | (<0.001) | (0.022) | (<0.001) |
| Observations | | 139,193 | 139,193 | 139,193 | 139,193 | 139,193 | 139,193 | 139,193 | 139,193 | 139,193 | 139,193 | 139,193 | 139,193 |

SEP, socioeconomic position. London*Intervention=post-intervention period in London (level), London*Intervention*Time=post-intervention trend in London (slope), London*Time=trend in London, Intervention*Time=post-intervention trend in the North of England. P-values in parentheses.
