# Supplementary material for: Changes in household food and drink purchases following restrictions on the advertisement of high fat, salt, and sugar products across the Transport for London network: A controlled interrupted time series analysis
Source: PLoS Med. 2022 Feb 17;19(2):e1003915. doi: 10.1371/journal.pmed.1003915 (PMC8853584; doi:10.1371/journal.pmed.1003915)
Supplement: S7 Table — (DOCX) [file pmed.1003915.s008.docx]

**S7 Table.** Coefficients for two-part model (packs).

|  | **Total HFSS** | | **Chocolate & Confectionery** | | **Puddings & Biscuits** | | **Sugary Drinks** | | **Sugary Cereals** | | **Savoury Snacks** | |
| --- | --- | --- | --- | --- | --- | --- | --- | --- | --- | --- | --- | --- |
| VARIABLES | Logit | NB | Logit | NB | Logit | NB | Logit | NB | Logit | NB | Logit | NB |
| **London* Intervention (level)** | 0.063 | 0.033 | 0.003 | 0.036 | -0.023 | -0.001 | 0.143 | -0.026 | 0.022 | 0.076 | 0.114 | -0.063 |
|  | (0.803) | (0.221) | (0.971) | (0.497) | (0.811) | (0.980) | (0.132) | (0.747) | (0.868) | (0.327) | (0.165) | (0.149) |
| **London* Intervention *Time (slope)** | -0.006 | -0.001 | -0.005 | -0.002 | -0.002 | 0.001 | -0.001 | 0.001 | 0.001 | -0.003 | -0.001 | 0.002 |
|  | (0.302) | (0.163) | (0.031) | (0.117) | (0.346) | (0.370) | (0.598) | (0.673) | (0.851) | (0.101) | (0.742) | (0.045) |
| Time | -0.005 | -0.004 | -0.007 | -0.005 | -0.008 | -0.002 | -0.007 | -0.002 | -0.000 | -0.001 | -0.004 | -0.003 |
|  | (0.255) | (<0.001) | (<0.001) | (<0.001) | (<0.001) | (<0.001) | (<0.001) | (0.176) | (0.824) | (0.587) | (0.006) | (<0.001) |
| London | -0.555 | -0.154 | -0.422 | -0.200 | -0.307 | -0.077 | -0.213 | 0.028 | -0.111 | 0.030 | -0.073 | 0.006 |
|  | (<0.001) | (<0.001) | (<0.001) | (<0.001) | (<0.001) | (0.004) | (0.001) | (0.566) | (0.135) | (0.478) | (0.183) | (0.840) |
| London*Time | 0.007 | 0.001 | 0.006 | 0.002 | 0.003 | -0.001 | -0.001 | -0.001 | -0.001 | 0.002 | -0.001 | -0.001 |
|  | (0.163) | (0.141) | (<0.001) | (0.043) | (0.132) | (0.137) | (0.769) | (0.598) | (0.609) | (0.111) | (0.723) | (0.285) |
| Intervention | -0.107 | -0.102 | 0.013 | -0.011 | -0.103 | -0.018 | -0.380 | -0.124 | 0.026 | -0.055 | -0.157 | -0.136 |
|  | (0.666) | (<0.001) | (0.866) | (0.827) | (0.245) | (0.597) | (<0.001) | (0.096) | (0.803) | (0.380) | (0.026) | (<0.001) |
| Intervention *Time | 0.006 | 0.005 | 0.006 | 0.004 | 0.008 | 0.002 | 0.010 | 0.003 | -0.002 | 0.001 | 0.005 | 0.004 |
|  | (0.215) | (<0.001) | (<0.001) | (<0.001) | (<0.001) | (0.003) | (<0.001) | (0.067) | (0.318) | (0.320) | (0.001) | (<0.001) |
| Weeks of Festival | -0.117 | 0.065 | 0.075 | 0.070 | -0.065 | 0.018 | 0.085 | 0.058 | -0.141 | -0.007 | 0.031 | 0.092 |
|  | (0.038) | (<0.001) | (<0.001) | (<0.001) | (0.001) | (0.029) | (<0.001) | (<0.001) | (<0.001) | (0.631) | (0.061) | (<0.001) |
| Number of Adults | 0.367 | 0.209 | 0.155 | 0.091 | 0.270 | 0.154 | 0.191 | 0.041 | 0.233 | 0.034 | 0.231 | 0.092 |
|  | (<0.001) | (<0.001) | (<0.001) | (<0.001) | (<0.001) | (<0.001) | (<0.001) | (0.032) | (<0.001) | (0.004) | (<0.001) | (<0.001) |
| Number of Children | 0.342 | 0.178 | 0.152 | 0.083 | 0.359 | 0.159 | 0.076 | 0.022 | 0.301 | 0.048 | 0.228 | 0.083 |
|  | (<0.001) | (<0.001) | (<0.001) | (<0.001) | (<0.001) | (<0.001) | (0.008) | (0.353) | (<0.001) | (0.001) | (<0.001) | (<0.001) |
| Seasons (Winter=0) |  |  |  |  |  |  |  |  |  |  |  |  |
| Spring | 0.044 | -0.087 | -0.014 | -0.093 | -0.083 | -0.068 | -0.074 | -0.012 | 0.068 | -0.007 | -0.075 | -0.071 |
|  | (0.671) | (<0.001) | (0.667) | (<0.001) | (0.023) | (<0.001) | (0.032) | (0.694) | (0.128) | (0.796) | (0.018) | (<0.001) |
| Summer | -0.063 | -0.118 | -0.194 | -0.168 | -0.086 | -0.066 | -0.123 | -0.009 | 0.087 | -0.003 | -0.111 | -0.092 |
|  | (0.403) | (<0.001) | (<0.001) | (<0.001) | (0.002) | (<0.001) | (<0.001) | (0.670) | (0.016) | (0.913) | (<0.001) | (<0.001) |
| Autumn | 0.022 | -0.062 | 0.126 | -0.023 | -0.011 | -0.028 | -0.114 | -0.045 | 0.009 | -0.011 | -0.080 | -0.084 |
|  | (0.683) | (<0.001) | (<0.001) | (0.059) | (0.609) | (0.001) | (<0.001) | (0.006) | (0.743) | (0.504) | (<0.001) | (<0.001) |
| Sex of main shopper (Female=0) | | | | | | | | | | | | |
| Male | -0.190 | -0.085 | -0.237 | -0.056 | -0.205 | -0.059 | -0.099 | -0.023 | -0.257 | -0.049 | -0.003 | 0.022 |
|  | (0.032) | (<0.001) | (<0.001) | (0.097) | (<0.001) | (0.033) | (0.077) | (0.568) | (<0.001) | (0.078) | (0.948) | (0.391) |
| Age of main shopper | 0.017 | 0.007 | 0.009 | 0.002 | 0.019 | 0.007 | 0.004 | 0.003 | 0.002 | 0.000 | -0.004 | -0.000 |
|  | (<0.001) | (<0.001) | (<0.001) | (0.023) | (<0.001) | (<0.001) | (0.032) | (0.017) | (0.385) | (0.847) | (0.025) | (0.607) |
| Socioeconomic position (High SEP=0) | | | | | | | | | | | | |
| Middle SEP | 0.213 | 0.116 | 0.191 | 0.071 | 0.221 | 0.092 | 0.142 | 0.052 | 0.060 | 0.025 | 0.152 | 0.068 |
|  | (0.018) | (<0.001) | (<0.001) | (0.021) | (<0.001) | (0.001) | (0.019) | (0.206) | (0.403) | (0.362) | (0.003) | (0.005) |
| Low SEP | 0.213 | 0.144 | 0.285 | 0.167 | 0.210 | 0.162 | 0.412 | 0.171 | -0.129 | -0.029 | 0.086 | 0.104 |
|  | (0.107) | (<0.001) | (<0.001) | (<0.001) | (0.004) | (<0.001) | (<0.001) | (0.001) | (0.168) | (0.329) | (0.227) | (0.004) |
| Constant | 2.078 | 1.896 | -0.597 | 1.017 | -0.538 | 0.661 | -1.504 | 0.493 | -2.451 | 0.255 | -0.300 | 0.768 |
|  | (<0.001) | (<0.001) | (<0.001) | (<0.001) | (<0.001) | (<0.001) | (<0.001) | (<0.001) | (<0.001) | (0.015) | (0.023) | (<0.001) |
| Observations | 139,193 | 139,193 | 139,193 | 139,193 | 139,193 | 139,193 | 139,193 | 139,193 | 139,193 | 139,193 | 139,193 | 139,193 |

NB, negative binomial. SEP, socioeconomic position. London*Intervention=post-intervention period in London (level), London*Intervention*Time=post-intervention trend in London (slope), London*Time=trend in London, Intervention*Time=post-intervention trend in the North of England. P-values in parentheses.
