# Supplementary material for: Changes in household food and drink purchases following restrictions on the advertisement of high fat, salt, and sugar products across the Transport for London network: A controlled interrupted time series analysis
Source: PLoS Med. 2022 Feb 17;19(2):e1003915. doi: 10.1371/journal.pmed.1003915 (PMC8853584; doi:10.1371/journal.pmed.1003915)
Supplement: S8 Table — (DOCX) [file pmed.1003915.s009.docx]

**S8 Table.** Difference in pre-intervention trends in energy (kcal) purchased from HFSS products in London (intervention) and the North of England (control).

|  | **Difference in pre-intervention trend,** Χ^2^ (P-value) |
| --- | --- |
| Total HFSS | 5.29 (0.07) |
| Chocolate & confectionery | **15.52 (<0.001)** |
| Puddings & biscuits | 2.55 (0.28) |
| Sugary drinks | 0.73 (0.69) |
| Sugary cereals | 0.27 (0.87) |
| Savoury snacks | 1.06 (0.59) |
| **Bold**, P<0.05. | |
