# Supplementary material for: Changes in household food and drink purchases following restrictions on the advertisement of high fat, salt, and sugar products across the Transport for London network: A controlled interrupted time series analysis
Source: PLoS Med. 2022 Feb 17;19(2):e1003915. doi: 10.1371/journal.pmed.1003915 (PMC8853584; doi:10.1371/journal.pmed.1003915)
Supplement: S9 Table — (DOCX) [file pmed.1003915.s010.docx]

**S9 Table.** Difference in pre-intervention trend for energy (kcal) purchased from chocolate & confectionery in London (intervention) and the North of England (control) using different start dates.

| **Week commencing** | **Difference in pre-intervention trend,** Χ^2^ (P-value) |
| --- | --- |
| 18^th^ Jun 2018 - original analysis | **15.52 (<0.001)** |
| 16^th^ Jul 2018 | **18.19 (<0.001)** |
| 20^th^ Aug 2018 | **18.72 (<0.001)** |
| 24^th^ Sep 2018 | **16.29 (<0.001)** |
| 01^st^ Oct 2018 | **13.66 (<0.001)** |
| 08^th^ Oct 2018 | **10.20 (0.01)** |
| 15^th^ Oct 2018 | **8.68 (0.01)** |
| 22^nd^ Oct 2018 | **8.80 (0.01)** |
| 29^th^ Oct 2018 | **6.25 (0.04)** |
| 05^th^ Nov 2018 | 4.26 (0.12) |
| 12^th^ Nov 2018 | **5.92 (0.05)** |
| 19^th^ Nov 2018 | 3.39 (0.18) |
| 26^th^ Nov 2018 | 0.57 (0.75) |
| 03^rd^ Dec 2018 | 0.43 (0.81) |
| **Bold**, P<0.05. | |
