# Supplementary material for: Changes in household food and drink purchases following restrictions on the advertisement of high fat, salt, and sugar products across the Transport for London network: A controlled interrupted time series analysis
Source: PLoS Med. 2022 Feb 17;19(2):e1003915. doi: 10.1371/journal.pmed.1003915 (PMC8853584; doi:10.1371/journal.pmed.1003915)
Supplement: S11 Table — (DOCX) [file pmed.1003915.s012.docx]

**S11 Table.** Changes in weekly household mean (95% CI) energy and nutrients purchased from chocolate and confectionery and packs of chocolate and confectionery purchased in London (intervention group) compared to the counterfactual using two pre-intervention periods.

|  | **Model 1** (main analysis) | **Model 2** |
| --- | --- | --- |
| Energy (kcal) | **-317.9 (-435.8 to -200.0)** | **-349.8 (-444.4 to -255.2)** |
| Fat (g) | **-13.1 (-18.8 to -7.5)** | **-12.5 (-16.7 to -8.2)** |
| Saturated fat (g) | **-8.7 (-11.7 to -5.7)** | **-8.2 (-10.4 to -6.0)** |
| Sugar (g) | **-41.4 (-55.4 to -27.4)** | **-49.0 (-60.5 to -37.4)** |
| Salt (g) | **-0.2 (-0.2 to -0.1)** | **-0.2 (-0.3 to -0.1)** |
| Packs (no.) | **-0.4 (-0.6 to -0.3)** | **-0.5 (-0.7 to -0.3)** |
| **Bold**, significant at 95% confidence level.  Model 1: Weekly household mean purchases estimated from controlled interrupted time series two-part model: part 1 (logit) and part 2 (generalised linear model) with gamma distribution for energy and nutrients and negative binomial distribution for packs. Models adjusted for festivals, season, number of adults in household, number of children in household, and sex, age and socioeconomic position of main food shopper. Cluster-robust standard errors used. Observations where households did not report any food and drink purchases that week were dropped. Data period=18 June 2018 to 29 December 2019.  Model 2: Model 1 using data period=19 November 2018 to 29 December 2019. | | |
