# Supplementary material for: Changes in household food and drink purchases following restrictions on the advertisement of high fat, salt, and sugar products across the Transport for London network: A controlled interrupted time series analysis
Source: PLoS Med. 2022 Feb 17;19(2):e1003915. doi: 10.1371/journal.pmed.1003915 (PMC8853584; doi:10.1371/journal.pmed.1003915)
Supplement: S13 Table — (DOCX) [file pmed.1003915.s014.docx]

**S13 Table.** Changes in weekly household mean (95% CI) energy (kcal) purchased from HFSS products, in London (intervention group) compared to the counterfactual, among households with no children and additional changes among households with children (n=1,970).

|  | **Change in energy purchased** | **Additional change in energy purchased** |
| --- | --- | --- |
|  | **No children (n=1395)** [reference group] | **Children (n=575)** |
| Total HFSS | **-1,261.6 (-1,886.3 to -636.9)** | 1020.7 (-224.1 to 2,265.6) |
| Chocolate & confectionery | **-244.3 (-380.5 to -108.2)** | -256.2 (-527.5 to 15.1) |
| Puddings & biscuits | **-287.1 (-511.0 to -63.3)** | 310.6 (-117.2 to 738.4) |
| Sugary drinks | 6.1 (-34.2 to 46.3) | 45.4 (-28.3 to 119.0) |
| Sugary cereals | -52.6 (-164.9 to 59.6) | 162.1 (-40.0 to 364.3) |
| Savoury snacks | 55.0 (-41.2 to 151.2) | 22.5 (-157.3 to 202.3) |
| **Bold**, significant at 95% confidence level. Weekly household mean purchases of energy from HFSS products estimated from controlled interrupted time series two-part model: part 1 (logit) and part 2 (generalised linear model) with gamma distribution. Models adjusted for festivals, season, number of adults in household, number of children in household, and sex, age and socioeconomic position of main food shopper. Cluster-robust standard errors used. Observations where households did not report any food and drink purchases that week were dropped. Data period=18 June 2018 to 29 December 2019. | | |
