# Supplementary material for: Changes in household food and drink purchases following restrictions on the advertisement of high fat, salt, and sugar products across the Transport for London network: A controlled interrupted time series analysis
Source: PLoS Med. 2022 Feb 17;19(2):e1003915. doi: 10.1371/journal.pmed.1003915 (PMC8853584; doi:10.1371/journal.pmed.1003915)
Supplement: S16 Table — (DOCX) [file pmed.1003915.s017.docx]

**S16 Table.** Changes in weekly household mean (95% CI) energy and nutrients purchased from HFSS products and packs of HFSS products purchased, in London (intervention group) compared to the counterfactual estimated using a mixed-effects negative binomial model (n=1,970).

|  | **Total HFSS** | **Chocolate & Confectionery** | **Puddings & Biscuits** | **Sugary Drinks** | **Sugary Cereals** | **Savoury Snacks** |
| --- | --- | --- | --- | --- | --- | --- |
| Energy (kcal) | **-1,220.8 (-1,785.0 to -656.5)** | **-391.5 (-534.1 to -248.1)** | **-327.8 (-548.0 to -108.7)** | -1.8 (-58.9 to 55.3) | -77,135.4 (-27,856.7 to 12,4296.9) | 98.7 (-25.2 to 222.5) |
| Fat (g) | **-76.8 (-113.9 to -39.8)** | **-16.7 (-23.9 to -9.4)** | **-13.7 (-23.8 to -3.6)** | -0.9 (-2.5 to 0.8) | -19.4 (-51.1 to 12.2) | 6.4 (-0.7 to 13.4) |
| Saturated fat (g) | **-31.8 (-46.2 to -17.3)** | **-10.4 (-14.1 to -6.7)** | **-5.6 (-10.8 to -0.5)** | -0.6 (-1.8 to 0.7) | -2.5 (-6.5 to 1.5) | 0.9 (-0.2 to 1.9) |
| Sugar | **-82.8 (-121.7 to -43.9)** | **-55.8 (-74.1 to -37.6)** | **-18.6 (-36.3 to -0.9)** | 3.4 (-6.8 to 13.6) | -73.5 (-306.3 to 159.3) | 1.0 (-0.3 to 2.4) |
| Salt | -3.2 (-9.2 to 2.8) | **-0.2 (-0.3 to -0.1)** | **-0.4 (-0.7 to -0.1)** | -0.0 (-0.1 to 0.1) | 0.0 (-0.2 to 0.3) | **0.4 (0.1 to 0.8)** |
| Packs (no.) | **-0.7 (-1.2 to -0.2)** | **-0.5 (-0.7 to -0.3)** | -0.11 (-0.29 to 0.07) | 0.0 (-0.1 to 0.1) | -0.0 (-0.1 to 0.0) | **0.1 (0.0 to 0.2)** |
| **Bold**, significant at 95% confidence level. Weekly household mean purchases estimated from a controlled interrupted time series mixed-effects negative binomial model. Models adjusted for festivals, season, number of adults in household, number of children in household, and sex, age and socioeconomic position of main food shopper. Cluster-robust standard errors used. Observations where households did not report any food and drink purchases that week were dropped. Data period=18 June 2018 to 29 December 2019. | | | | | | |
