# Supplementary material for: Changes in household food and drink purchases following restrictions on the advertisement of high fat, salt, and sugar products across the Transport for London network: A controlled interrupted time series analysis
Source: PLoS Med. 2022 Feb 17;19(2):e1003915. doi: 10.1371/journal.pmed.1003915 (PMC8853584; doi:10.1371/journal.pmed.1003915)
Supplement: S1 Text — (DOCX) [file pmed.1003915.s022.docx]

**S1 Text.** Study protocol

**Restricting advertising of high fat, salt and sugar foods and drinks on the Transport for London network: evaluation of a natural experiment**

ISRCTN19928803 <https://doi.org/10.1186/ISRCTN19928803>

### **PLAIN ENGLISH SUMMARY**

**Background and study aims**
Marketing and branding are effective in encouraging the purchase and consumption of unhealthy food and drink. Advertising impacts children’s food choices and results in pressure being put on parents to buy unhealthy foods. The Mayor of London has therefore restricted the advertising of less healthy high fat, sugar and salt (HFSS) foods and drinks across the Transport for London (TfL) estate. This new policy came into force on 25th February 2019.
This study aims to evaluate if the removal of HFSS advertising changes people’s exposure to HFSS advertising, changes people’s awareness of HFSS foods and drinks and changes household purchasing of HFSS products. It will also explore how the policy was developed and implemented.

**Who can participate?**
Members of public aged 18+, as well as key stakeholders working in public health, local government, and the food and marketing industry.

**What does the study involve?**
To establish if the policy has an impact on the purchase of HFSS foods and drinks in London, we will use data provided by the Kantar WorldPanel household consumer panel. Households are recruited to the panel to provide data on their day-to-day food and beverage purchases. A short survey will be sent out to members of the panel to determine if there are any changes to perceptions and awareness of HFSS foods and drinks. We will also explore changes in exposure to HFSS advertising using data provided by TfL such as the location and content of advertisements. Finally, we will carry out interviews and focus groups with the public and key stakeholders to explore their views and reactions to the restrictions of HFSS advertising across the TfL estate.

**What are the possible benefits and risks of participating?**
The information participants provide will help to inform national and local policies on HFSS advertising, which may be an indirect benefit.

**Where is the study run from?**
The study is being led by the London School of Hygiene and Tropical Medicine and alongside collaborators from the Universities of Bristol, Cambridge, Liverpool and Teesside.

**When is the study starting and how long is it expected to run for?**
1st January 2019 to 31st March 2021

**Who is funding the study?**
National Institute for Health Research School for Public Health Research (UK)

**Who is the main contact?**
Professor Steven Cummins: steven.cummins@lshtm.ac.uk

### **Trial website**

### <https://sphr.nihr.ac.uk/>

## **CONTACT INFORMATION**

### **Type**

### Scientific

### **Primary contact**

Prof Steven Cummins

### **ORCID ID**

<http://orcid.org/0000-0002-3957-4357>

### **Contact details**

Department of Public Health
Environments & Society
Faculty of Public Health & Policy
London School of Hygiene & Tropical Medicine
15-17 Tavistock Place
London
WC1H 9SH
United Kingdom
+44 (0)20 7636 8636
[Steven.cummins@lshtm.ac.uk](mailto:Steven.cummins@lshtm.ac.uk)

## **ADDITIONAL IDENTIFIERS**

### **Protocol/serial number**

SPHR-PROG-PCBF-WS1WP1

## **STUDY INFORMATION**

### **Scientific title**

Restricting advertising of high fat, salt and sugar foods and drinks on public transport networks in London: evaluation of a natural experiment to assess impacts on awareness and purchasing of foods high in salt, fat and sugar

### **Study hypothesis**

The Mayor of London has implemented restrictions on the advertisement of high fat, sugar and salt (HFSS) foods and drinks across the Transport for London (TfL) estate in order to help tackle childhood obesity. Advertising on the TfL estate makes up approximately 40 per cent of London’s outdoor advertising by revenue. This new policy came into force on 25th February 2019. This study aims to evaluate the impact of these restrictions on peoples’ awareness of HFSS foods and beverages, household purchasing of HFSS foods and beverages; as well as to understand the genesis of the policy and how it was developed and implemented.

### **Ethics approval**

Approved 11/01/2019 and 29/5/2019, the London School of Hygiene and Tropical Medicine Ethics Committee (Keppel Street, London, QC1E 7HT; ethics@lshtm.ac.uk; <http://www.lshtm.ac.uk/ethics>/), ref: 16297 /RR/11721 and ref: 16297 /RR/14307.

### **Study design**

Mixed-methods evaluation of a natural experiment comprising of (i) an impact evaluation on household purchasing behaviour that utilises a controlled interrupted time-series design and (ii) a process and implementation evaluation that utilises realist principles.

### **Primary study design**

Observational

### **Secondary study design**

mixed-methods evaluation

### **Trial setting**

Other

### **Trial type**

Prevention

### **Patient information sheet**

Not available in web format, please use contact details below to request a participant information sheet.

### **Condition**

Children’s food preferences, purchasing requests, purchases and consumption

### **Intervention**

The Mayor of London has implemented restrictions on the advertising of high fat, sugar and salt (HFSS) foods and beverages across the TfL estate. These restrictions prevent the advertising of products considered HFSS across all outdoor advertising media (e.g billboards, screens and other media), excluding incidental advertising where the product is not the main focus of the advert. The categorisation of products as HFSS is determined using the Public Health England (PHE) Nutrient Profiling Model where a score of 4+ for foods and 1+ for drinks classifies the item as an HFSS product. Companies and brands can apply for an exemption to this restriction if they can prove that the item is not consumed or marketed to children under the age of 18. The intervention came into force on 25th February 2019.

We aim to undertake a mixed-methods evaluation comprising: (i) an impact evaluation of changes to awareness of HFSS foods and drinks, and household food HFSS purchasing and (ii) a process and implementation evaluation.

Impact Evaluation
The impact evaluation will utilise secondary data from public and commercial providers primarily obtained from the Greater London Authority (GLA), Transport for London (TfL) and Kantar WorldPanel (KWP).
1. TfL in collaboration with JC Deceaux and Exterion Media will provide secondary data that will allow geocoding of the location of advertising sites together with metadata on the purchaser (brand/company) and duration of campaign for that purchaser. These data will be linked using postcode district to household level KantarWorldPanel data.
2. KantarWorldPanel will provide item-level longitudinal time series data on ‘take-home’ and ‘out-of-home’ food and beverage purchases from their nationally representative continuous household consumer purchase panel. Households were recruited from London (intervention region) and North East England (control region) and provide product item-level data collected using scanners. The two regions were matched in terms of underlying socio-demographic composition of the sample.
3. Within the KantarWorldPanel panel we commissioned a short (15 minute) online longitudinal questionnaire survey of the main food purchaser in each of the recruited households in both London and the North East. The survey collected data on perceived exposure and awareness of HFSS product advertising (both within and outside of the TfL network). These data will then be linked to households’ item-level data of household food and beverage purchasing.

Data will be analysed using (i) a controlled interrupted time-series (ITS) design to explore impacts on household purchasing of HFSS foods and beverages and (ii) a difference-in-difference analyses for perceived exposure and awareness of HFSS advertising. The ITS analysis will explore impacts using item-level data for aggregated food categories (e.g sugar-sweetened beverages, sweet snacks and confectionary) and specific product groups (e.g chocolate) and the initial proposed impact model will assess whether there is an immediate level and slope change given the specific implementation date. We will also explore impacts on objective and perceived exposure to advertising and investigate any substitution effects. Where possible, we will explore whether any observed effects are moderated by household socio-economic status and baseline HFSS purchasing.

Process & Implementation Evaluation
We will conduct a qualitative process and implementation evaluation drawing on the MRC Process Evaluation Guidance. The evaluation will comprise:

1. Documentary analysis of materials generated from the GLA and TfL related to the development and implementation of the HFSS advertising ban, such as meeting and committee minutes and reports. This will include material from the joint GLA/TfL Exceptions and Policy panels.
2. In-depth interviews and focus groups with a range of key stakeholders from GLA, TfL and Industry (advertisers, companies, brands and marketeers) and the public (detailed below).

In-depth interviews: Elite interviews will be carried out with key stakeholders, and decision-makers in the GLA, TfL, and companies that manage advertising for TfL to explore narratives of the development and implementation of the ban and the values underpinning it.

Expert interviews will be carried out with commercial/corporate stakeholders in order to identify, contextualise and critique existing and potential food industry responses to the ban.

Focus groups: Focus groups will be carried out with adults living and working in London. This will allow in-depth investigation of perceptions of the ban and reactions to it at the household level.

Interviews and focus groups will be transcribed verbatim, uploaded to NVivo software and subjected to a General Inductive Approach (GIA) analysis. Complete texts from documents and interviewers’ notes will be uploaded into NVivo for analysis alongside transcripts of interviews and focus groups.

### **Intervention type**

Other

### **Primary outcome measure**

1. Changes in exposure to HFSS food and beverage advertising at the food and beverage category level is measured using site-level objective advertising data at 12 months post-implementation of the intervention.
2. Changes in adult-reported perceptions and awareness of HFSS food and beverages amongst adults and children is measured using a questionnaire at 12 months post-implementation of the intervention.
3. Changes in household purchases of HFSS foods and beverages at the product category and item level from households is measured using KantarWorldPanel data (product item-level scanner data) at 12 months post-implementation of the intervention.

### **Secondary outcome measures**

1. Substitution effects towards other foods are measured using KantarWorldPanel data at 12 months post-implementation of the intervention.
2. Whether any observed effects in primary outcomes and secondary are moderated by socioeconomic status, education, presence of children and baseline purchasing of HFSS foods (low, medium and high) are measured using KantarWorldPanel data at 12 months post-implementation of the intervention.

### **Overall trial start date**

01/01/2019

### **Overall trial end date**

31/03/2021

## **ELIGIBILITY**

### **Participant inclusion criteria**

KWP HFSS food and beverage purchase and Surveys on perception and awareness of HFSS:
1. Aged 18 and over.
2. Part of the Kantar Worldpanel household consumer panel.

Interviews:
Key stakeholders, and decision-makers in the GLA and TfL, and commercial/corporate stakeholders in the food and marketing industry.

Focus group:
1. Aged 18 and over.
2. Living and working in London.

### **Participant type**

Mixed

### **Age group**

Adult

### **Gender**

Both

### **Target number of participants**

HFSS food and beverage purchase: A sample of n=1400 households will be selected from the (demographically representative) London Regional Sample (n=700) and a non-London comparator sample (North East England) that excludes London Commuters (n=700) of the KantarWorldPanel household panel (UK n=32,000). Surveys: Approximately n=2000 households will be invited to achieve responses from n=1400 households (based on an estimated 70% response rate). In-depth interviews: Approximately purposively selected 15 key stakeholders, and decision-makers in the GLA and TfL, and 5-10 key commercial/corporate stakeholders in the food and marketing industry. Focus groups: We will recruit 3 focus groups with approximately 6-8 participants per focus group (n= 24 participants). Focus groups will be sampled for diversity in terms of socio-demographic characteristics and include households with and without children.

### **Participant exclusion criteria**

N/A

### **Recruitment start date**

08/02/2019

### **Recruitment end date**

30/09/2019

## **LOCATIONS**

### **Countries of recruitment**

England, United Kingdom

### **Trial participating centre**

London School of Hygiene and Tropical Medicine
15-17 Tavistock Place
London
WC1H 9SH
United Kingdom

## **SPONSOR INFORMATION**

### **Organisation**

London School of Hygiene and Tropical Medicine

### **Sponsor details**

Keppel Street
London
WC1E 7HT
England
United Kingdom
+44 (0)20 7636 8636
[study@lshtm.ac.uk](mailto:study@lshtm.ac.uk)

### Sponsor type

University/education

### Website

<https://www.lshtm.ac.uk/>

### **GRID**

[grid.8991.9](https://www.grid.ac/institutes/grid.8991.9)

## **FUNDERS**

### **Funder type**

Government

### **Funder name**

National Institute for Health Research School for Public Health Research

## **RESULTS AND PUBLICATIONS**

### **Publication and dissemination plan**

We expect to produce peer-reviewed publications in high impact journals from the study. In addition, we expect to produce briefings (both oral and written) for the Greater London Authority and develop guidance for other local authorities thinking of implementing similar bans in collaboration with the GLA. Work will also be presented at conferences/events targeted at various stakeholders and the general public.

### **Intention to publish date**

31/03/2022

### **Individual participant data (IPD) sharing statement**

Data was purchased from a commercial provider (Kantar WorldPanel) and the terms of our contract states that data can only be used by the named research team, therefore no data can be shared.

### **Participant level data**

Not expected to be made available
