# Supplementary material for: Changes in household food and drink purchases following restrictions on the advertisement of high fat, salt, and sugar products across the Transport for London network: A controlled interrupted time series analysis
Source: PLoS Med. 2022 Feb 17;19(2):e1003915. doi: 10.1371/journal.pmed.1003915 (PMC8853584; doi:10.1371/journal.pmed.1003915)
Supplement: Attachment — Submitted filename: TfL_impact_ResponsetoReviewers_02Dec2021.docx [file pmed.1003915.s024.docx]

Requests from the editors:

Please restructure the abstract so that the final sentence of the "Methods and findings" subsection begins "Study limitations include ..." or similar and quotes 2-3 of the study's main limitations.

We have restructured the Methods & Findings section so that the final sentence describes study limitations as follows: “Study limitations include the lack of out-of-home purchases in our analyses and not being able to assess the sustainability of observed changes beyond 44 weeks.”

Please add a sentence to the abstract around line 44 to quote the baseline differences in the samples (information around line 274 in the Results section).

We have added a sentence about the sociodemographic differences between the samples as follows: “The intervention and control samples were similar in household characteristics but had small differences in main food shopper sex, socioeconomic position and body mass index.”

Please avoid claims such as "the first" at line 57, and where needed add "to our knowledge" or similar.

We have removed this claim.

After the abstract, please add a new and accessible "Author summary" section in non-identical prose. You may find it helpful to consult one or two recent research papers in PLOS Medicine to get a sense of the preferred style.
We have added an Author Summary.

Noting the study registration, is a protocol or prespecified analysis plan available? If so, please attach this as a supplementary file, referred to in the Methods section.

The protocol is linked on line 151 in the Methods and now also provided as Supplementary Text S1. There was no separate analysis plan.

Please highlight non-prespecified analyses.

Several sensitivity analyses were conducted to test the robustness of our findings. These include: analysing a sub-sample of regular reporters; using a mixed-effects negative binomial model for methodological triangulation; moving the modelled intervention date to test the specificity of the findings to time of implementation; examining changes in purchases by public transport use; and restricting the time series to remove the Christmas period at the end of the study period. These are all described and highlighted in line 279-305. In response to reviewer comments, we now also explore changes in non-HFSS purchases (lines 307-309) and compare the changes in the first and last post-intervention weeks (lines 263-266).

Please avoid language implying causality, throughout the text. For example, at line 54 in the abstract "observed effects" should be amended to "observed changes" or similar. Similarly, please adapt the wording to "Relative reductions ..." or similar at line 49, for example.
We have edited the wording throughout to avoid language that implies causality and to make it clear that the changes detected were relative to the counterfactual.

Throughout the text, please locate reference call-outs prior to punctuation, e.g., "... HFSS products [1,2,4].".

Amended.

Please remove the information on data sharing and funding from the end of the main text. In the event of publication, this information will appear in the article metadata, via entries in the submission form.
Removed.

In the reference list, please use the journal name abbreviations "PLoS Med." and "PLoS ONE".
We have changed all incidences of “PLOS Med” to “PLoS Med” and “PLoS One” to “PLoS ONE”.

For reference 3 and the other relevant citations, please remove the name of the paper's academic editor.

All mentions of academic editor removed.

Please include a completed STROBE checklist with your revision, labelled "S1_STROBE_Checklist" or similar and referred to as such in the Methods section (main text).

In the checklist, please refer to individual items by section (e.g., "Methods") and paragraph number, not by line or page numbers as these generally change in the event of publication.
We have attached a STROBE checklist (Supplementary Text S2) with our revised submission and referred to it in the Methods section (line 153).

Comments from the reviewers:

*** Reviewer #1:

Thanks for the opportunity to review your manuscript. My role is as a statistical reviewer so my review focuses on the study design, data, and the analysis that is presented. I have put general queries first and followed those with questions relevant for a specific section of the manuscript.

This study uses data from a commercial panel study, and includes households in London and the north of England. The event of interest was the introduction of new advertising policy on the public transport system in London (TfL). The time series included 36 weeks of data before the event change in advertising, and 44 weeks after the event. A hurdle model (logit - bought item, GLM - how much purchased). The strengths of the study are the control region, a regression model well-suited to an 'energy purchased' variable, and measurements very specific to what the policy change aimed to achieve. The manuscript is well-written and has well-presented tables and figures and thoughtful sensitivity analyses.

We thank the reviewer for comments on our work. We are glad that the reviewer finds our study design, use of control and sensitivity analyses to be strengths of our work and that the manuscript is well-written.

There are some details in the pre-registered protocol, as a more detailed statistical analysis plan developed for the controlled ITS? The protocol also details a qualitative and impact evaluation - are these currently under review elsewhere?

We did not submit a separate statistical analysis plan. We have published an analysis of media coverage of opposition to the ban (Thompson et al 2021: <https://doi.org/10.1016/j.ssmph.2021.100828>) and a process and implementation study (Meiksin et al 2021: <https://doi.org/10.1016/j.socscimed.2021.114548>). These are now cited in the manuscript on lines 140-141.

Unfortunately, some analyses mentioned in the protocol are now not possible. We were unable to assess changes in advertising exposure and awareness, as the second (post-intervention) survey was completed in March 2020, and therefore was contaminated by changes in transport use due to the COVID-19 pandemic. Data on the location and content of TfL advertisements were ultimately unavailable from commercial organisations, despite a lengthy negotiation process.

P2, L42. Could you clarify what each n refers to here? I.e. is the first n the number of records? The number of participants might be more useful to include.

The first n referred to the number of food and drink purchases. We have now reworded this sentence to make this clearer (see line 41). n=977 and n=993 refer to the number of households included in the study from the intervention and control area, respectively.

P2, L46. I would drop the 'n=' from the study periods here and on line 47
Dropped.

P3, L91. Could you briefly describe what the 'TfL' network composes? I assume this includes advertising at train stations, bus shelters, and advertising on the public transport vehicles as well.
The TfL advertising estate includes the London Underground network, the TfL Rail network, transport vehicles run by TfL (including some buses, trains and taxis) and outdoor spaces owned by TfL (e.g. bus stops and land outside train stations). We have added this information on lines 130-132.

P5, L121. Which areas in the 'North of England' were sampled? Can the postcodes or areal identifiers be included as an appendix?

The North of England sample consisted of households from the North West, North East, and Yorkshire and the Humber regions. We have now added this information to the manuscript (lines 163-164) and included a map highlighting the intervention and control areas (see Figure 1).

P5, L124. Perhaps worth being specific and describing it as a 'location-based control'?

Thank you for this suggestion. We have done this on line 165.

P5, L125. So some households did not contribute data to the entire time-series? How many households had complete data across the study period?

On average, household contributed data to 70.7 of the 80 study weeks (see line 183). Overall, 1126 (57.2%) households reported in more than 72 (90%) of the study weeks (see line 280-283), with 209 (10.6%) reporting in all 80 weeks, 172 (8.7%) in 79 weeks and 118 (6.0%) in 78 weeks. However, not purchasing in a given week could be for a variety of reasons, including that households do not habitually shop weekly (see lines 183-185). Therefore, it is not necessarily the case that only households that reported purchases in all 80 weeks have complete data.

P5, L127. 'global seasonal fluctuation' might be a better description as it's impossible to exclude a seasonal effect unique to one of the two study locations.

We have rephrased this sentence for clarity (lines 167-169).

P6, L191. Is this the Huber 'sandwich' robust standard error?
We included Huber/White/sandwich estimators (using the robust option for the *twopm* command in Stata) and clustered sandwich estimators (using the cluster option for the *twopm* command in Stata). Descriptions are provided in the following Stata manuals: <https://journals.sagepub.com/doi/pdf/10.1177/1536867X1501500102> and <https://www.stata.com/manuals/xtvce_options.pdf>.

P8, L221. Just to check - these are from the 'margins' command in Stata? For my own interest - which Stata command did you use to estimate the two-part model that was compatible with margins?

Yes, we used the *margins* command in Stata following the user-written *twopm* command for the two-part model.

Fig 2. Fascinating figures - it turns out my vision of the English tucking into pudding and chocolates over the chilly Christmas period is completely correct. The line for the counterfactual seems to get lost in this panel (e.g. sugary cereals/chocolate). I can't give you a specific recommendation to improve this but perhaps it's worth taking a second look at these figures?

We are now using brighter colours and thinner lines in the Figure and have provided a higher resolution file. We hope that this improves the visibility of the counterfactual line.

Supp Table 12. Where these estimated by fitting a separate model for each sub-group? One limitation of this approach is that you shrink the sample size and so whether specific subgroup has a 'significant' treatment effect then depends strongly on how much sample size there is in it (and relies on 'differences in nominal significance'). An alternative is to include the sub-group variable in the regression model and look at the interaction of this with the relevant parameters to test for overall heterogeneity. I have to admit I am not sure how or if this could be achieved easily with the two-stage model you are using. You are fairly careful about describing these analyses but I would consider being clear about the limitation of a stratified analysis rather than one with an interaction to look for sub-group heterogeneity.

Thank you for this comment. Yes, we fitted a separate model for each subgroup and agree that this method is limited by the small sample sizes. We now use interaction terms to investigate sub-group differences (see lines 268-273 and 397-406) in order to maximise the available sample size.

*** Reviewer #2:

This study evaluated the implementation of an intervention to restrict advertisement of HFSS products in public transport using household purchasing data. The paper is well written although there are several points which need clarification. A major concern is the comparability of the intervention and control sample, especially given the absolute differences in baseline purchasing patterns between the samples.

Thank you for reviewing our work. A controlled ITS approach requires parallel trends in HFSS purchasing over the pre-intervention period between the intervention and control regions as a standard assumption, in order to control for time-varying confounders e.g. other events occurring in both areas around the time of the intervention (<https://academic.oup.com/ije/article/47/6/2082/5049576>). This differs from some other study designs where the control is used as the sole means of approximating the counterfactual (e.g. RCTs and controlled before-after studies) and similar ‘levels’ in the control and intervention areas are required. The parallel trends assumption is met in the analyses (see lines 345-352), and therefore, a controlled ITS approach is the appropriate analytical design for our data. For chocolate and confectionery, where the parallel trend assumption was met only when earlier time points were excluded, we undertake further sensitivity analyses to test the robustness of our findings (see lines 374-375).

Specific comments:
Introduction
Line 84-88 Can the authors describe what other locations they mean here? In general there's enough background on the HFSS on TV but no much on public transport or other relevant environments

We have clarified that we mean other countries and regions (line 121). To our knowledge, there has only been one study that evaluated advertising restrictions outside of TV advertising restrictions. We mention this evaluation of Quebec’s advertising restrictions in the Discussion (lines 486-487) and have now provided some details on this study in the Introduction (lines 124-126).

Methods
Line 125-128 How comparable is the control sample of households in terms of exposure to advertisement through public transportation? A bit more description of this is needed. As seen in Box1, TfL is a very large network which means people may be highly exposed to advertisements whereas in the control area this may not be the case, perhaps limiting the comparability of the chosen sample?
Are there any other criteria for matching (if this was done) intervention and control households?

In our previous work (<https://bmjopen.bmj.com/content/11/4/e048139.info>), we found that 45.5% of London participants and 28.8% North of England participants reported seeing HFSS advertising on a transport network in the last 7 days. This survey was conducted in February 2019, but prior to the advertising restrictions. We now mention this on lines 336-338. We found differences in public transport use in this study, as expected of the regions (<https://www.statista.com/statistics/682749/passenger-transport-service-in-the-united-kingdom-uk-by-region/>) (see lines 333-336). We assume that the difference in HFSS advertising exposure in the two regions remains constant over the study period, except for a reduction in HFSS advertising in London, relative to the North of England, in the post-intervention period as a result of the TfL restrictions.

The difference in HFSS advertising exposure does not limit the comparability of the samples, as our study design is based on the assumption that the trend in outcome (i.e. purchases) is parallel over the pre-intervention period (see above), rather than having similar level of exposure. This is the standard approach in CITS studies. We tested for parallel pre-intervention trends in HFSS purchases, finding only chocolate and confectionery had deviations from the parallel trend (see lines 346-352). We do an additional sensitivity analysis for chocolate and confectionery for this reason (see lines 374-375). We are, therefore, confident that the North of England is a suitable control in our study and helps to control for background trends in HFSS purchasing (i.e. increases over time) and seasonal fluctuations that are common to both areas when used to construct our counterfactual.

The two regions in the study were selected to be broadly comparable on the basis of household characteristics of panel members by Kantar. Households within each region were then randomly sampled.

Line 140 Can the mean study weeks include the IQR (instead/in addition to SD)

We have now provided the mean, SD, median and IQR on line 183.

Line 144 Can the % missing be reported here

Weeks with no purchases account for 11.7% of all the data. We have now stated this on line 187. However, as we mentioned above (page 3), no purchases in a given week does not necessarily indicate missing data, as some households will habitually shop less than once a week.

Line 151-152 Can the authors estimate the % imputed values in this study - instead of referencing other studies?

This data was unfortunately not available to us. However, we do not have any reason to believe that the % of imputed values would differ substantially from earlier periods and provide the values for years 2012-2017 as an indication (these ranged from 11.0% for energy and 19.6% for fibre).

Line 166 Why is the variable for public transport use (yes/no) used in a sensitivity analysis, rather than a secondary or exploratory outcome? Was this not part of the initial protocol?

This sensitivity analysis was not included in the initial protocol, but we believe that it strengthens our case for the observed changes being related to the TfL policy, as regular public transport users were likely to have had larger changes in their HFSS advertising exposure as a result of the TfL policy. As mentioned above, our plan to measure change in advertising exposure through surveys was impacted by the pandemic, and therefore, this analysis could not be conducted. Transport use (as an outcome) is unlikely to plausibly change as a result of the implementation of TfL advertising policy and, for this reason, we did not consider public transport use as an outcome.

Line 174 The description of the main nutrient outcomes could be clearer. Is this kcal from HFSS/household/week?

Yes, we have now clarified this (see lines 222-224).

Have the author considered using mean food expenditure/week as an additional outcome?

We did consider using food expenditure as an outcome, however, felt that this would be difficult to interpret due to the range in prices of products. Purchasing more expensive or cheaper products as a substitute does not indicate the healthiness of the swap. Therefore, we did not include expenditure as an outcome, given that we have already included various outcome measures: energy, nutrients and packs.

Statistical analysis. The choice of models and sensitivity analyses seem robust. However, I wonder why the authors haven't included an analysis using monthly purchases instead of weekly. I think that there could be a period of time (perhaps several weeks) from intervention implementation at which effects are detectable. Perhaps for something like advertisement, removal of HFSS ads may not have an immediate effect on purchases and a longer period of time may need to be considered.

We used weekly rather than monthly purchases in order to preserve statistical power. As our analysis estimates the average weekly purchases over the post-intervention period, any changes throughout the post-intervention period are taken into account and there should be no additional change that would be detected by aggregating the data monthly rather than week. However, we see from Figure 3 that the difference between the observed and counterfactual values get larger over time. We now also compare the changes detected in the first and last post-intervention weeks (see Supplementary Table S10) and observe larger relative reductions in the last week (see lines 361-363). This could be, as you suggest, due to changes taking some time to occur or to be detectable. We have added a comment to that effect in the Discussion (lines 526-532).

Results
Table 2 doesn't seem to be sufficiently described in the manuscript. There are absolute differences before policy implementation between intervention and control samples, especially for chocolate & conf. Where these baseline purchasing patterns tested? Large differences between intervention and control samples may limit their comparability.

Table 2 is now described more fully in the text (see lines 342-345). There are absolute differences between the two samples, but the difference (which is controlled for in the model via an indicator variable for the region) is consistent over time before the introduction of the policy. This was tested using significance of the London*time interaction term in our models (see lines 345-352). There were some deviations from the baseline difference detected for chocolate and confectionery in the earlier weeks, but we re-ran the analysis using only the pre-intervention period where trend in purchasing was parallel, and obtained similar effect sizes (see lines 374-375). As mentioned above, our study design does not require the same level of purchasing in the intervention and control areas in the pre-intervention period, but rather an assumption of a parallel trend in purchasing between the two areas in the pre-intervention period.

Results/discussion
In general, the authors talk about the "reduction" in HFSS observed after the intervention implementation, whereas looking at the trends, it feels like it's rather an attenuation of the growth that would be expected in normal circumstances. I would advise to revise the text accordingly

We agree that it is an attenuation of growth rather than absolute reduction and discuss this in the Discussion section (see lines 432-436 and line 542). We compare the observed purchases with the counterfactual throughout the manuscript and have gone through the text, and revised where necessary, to make sure this is clearly communicated. As recommended by the editor, we now use ‘relative reduction’ throughout the manuscript.

Is there an explanation for the results observed in sugary drinks/cereals and savoury snacks?
As described in the manuscript, the policy itself is focused on product advertisement only, this means that brand advertising continues for those products with a policy compliant alternate product (i.e. a lower or zero calorie version) and therefore may have contributed to maintaining sugary drink, sugary cereal and savoury snack purchases. These HFSS categories often have non-HFSS products that could have been advertising on the TfL estate. We have expanded our discussion of this (see lines 506-524).

*** Reviewer #3:

General Comments: The study evaluates whether purchases of typically HFSS products improved in nutritional measures before vs after the TfL's advertising policy was implemented in Feb 2019 focusing on Londoners' (and also Londoners' who reported using TfL) vs a comparison group who should have limited exposure to the TfL policy. The combination of both a traditional DID design (given the control group) and a counterfactual design is confusing given that the results focused on the counterfactual approach. The authors need to be a better job explaining their choices. The study is an important one and can add significantly to the literature around unhealthy food marketing and regulations that need to work in concert to minimize it.
We thank the reviewer for recognising the importance of our work and for their comments on our manuscript. Whilst a CITS and DiD approach would have been possible, we believe that a CITS design has advantages for our particular study (<https://academic.oup.com/ije/article/47/6/2082/5049576>). Our approach allowed for the intervention effect to change over time. By modelling the underlying trend, ITS controls for within-group characteristics that tend to change only slowly over time, secular changes, random fluctuations from one time point to the next and regression to the mean. Furthermore, as our evaluation is based on observing a single population (i.e. London households) over time, the CITS design is free from problems due to between-group differences, such as selection bias or unmeasured confounders. We have elaborated on our reasoning in responses to comments below.

Specific Comments

1) Abstract Line 45: should be "energy and nutrients".
Corrected.

2) It might have been informative for the study to also have included a non-HFSS food category as a potential control food category to see if those trends were parallel for Londoners vs North Englanders. Alternatively it would have been useful to see what energy purchased from non-HFSS categories might have changed (or not).

Thank you for this suggestion. We have now included an analysis of changes in non-HFSS purchases (see lines 307-309; 416-419, Table 2 and Supplementary Table S20). No significant changes in energy or nutrients purchased from non-HFSS products, or number of packs of non-HFSS products purchased, were detected.

We did consider a control food category, however, we rejected this on the basis that the policy may stimulate a switch into another (unknown) product category. We could also have used a non-equivalent control (such as toiletries), however, these data were not available to the research team.

3) Why didn't the authors consider using Feb 2018-Nov 2018 as the "pre" period and Feb 2019-Nov 2019 as the "post" period? Is there reason to think that the 3 months between the announcement and implementation would have had some impact on the outcomes? This also helps deal with potential seasonality issues. Or was the choice to only start later in 2018 related to wanting to avoid the SDIL implementation? If so be explicit.

Unfortunately, we only had data from June 2018 to March 2020 available to us. The announcement was unlikely to impact on changes in exposure as the long lead in time for commercial advertising and the duration of existing campaigns meant that existing campaigns persisted up until the implementation date. We undertook temporal falsification in our sensitivity analyses. As we have reported, here the changes observed in this study were robust to the date of implementation (see lines 411-413).

4) It might be useful to provide a map to illustrate the boundaries and areas of residence among the sample used for the "treatment" vs " control" households.
Thank you for this suggestion. We have now included a map highlighting the intervention and control areas (see Figure 1).

5) Pls provide more information on how the authors determined the NPM scoring, particularly around FV content. What approximations did they have to use given that this information is not easily available?

Fruit and vegetable content was estimated by Kantar, with product categories categorised as High, Mix or Low in fruit, veg and nuts. For the NPM calculation, high was treated as 5 points/>80% FVN content, Mix as 1 points/40% FVN content, and Low as 0 points/≤40%. We have now included this information in the manuscript (lines 217-220).

6) The authors state that they were unable to assess volume purchased. How did then they obtain information about the total energy and nutrients purchased from the HFSS categories?

Was the nutrient information available already able to account for volume? Typically the nutrient information is in the form of nutrient densities (per 100g or per 100ml), which then allows for "copying" of nutrient density values across products (adjusting for volume or package sizes), as well as for the application of the NPM. As such, it is unclear how the nutrient values are derived and why there aren't also volume measures, especially if package sizes can vary considerably.

Energy and nutrient data are indeed usually provided per 100 g or per 100 ml. Kantar provided data on the energy and nutrients content per purchase. They also provided a volume measure for each purchase. Most volume measures were in kg or L, but some products had units or packs as their volume measure, which made them incomparable.

To determine whether a purchase was HFSS or not, we used that volume measure to obtain energy and nutrients per 100 g. Where products were classified in units/packs, we did manual adjustments. Some categories could be reclassified as a whole category – e.g. all chocolate products were re-classified as HFSS. This is because chocolates have a minimum required saturated fat content that makes it extremely difficult for chocolate to be non-HFSS. Some types of biscuits, cakes and puddings were also manually changed to HFSS, whilst all fruit and vegetable were reclassified as non-HFSS.

We are confident that any bias would be small as 95% of observations were correctly specified, as they were not measured in units. Further, where products were measure in units/packs, some whole categories could be classified as non-HFSS or HFSS. However, the volume measure itself is not reliable enough to use as an outcome measure, and particularly for chocolate and confectionery where there were a large number of purchases with a volume measure of packs rather than weight, which is why we were unable to assess volume purchased.

7) Was the nutrient information updated within the time period of the data used in the analyses? Or was it the same throughout the period? It would be useful to be explicit about this because then it provide an indication as to whether some potential reformulation may have happened (or not).

Nutrient data is collected twice a year by Kantar (see line 191-192). Over the study period, a small number of nutrient values may have changed for specific products due to ongoing reformulation. However, it is highly unlikely that there would be differential reformulation by region given the way products are manufactured and produced and the costs involved. Given our outcome is a relative reduction in energy and nutrients, the fact that there will be no systematic differences in reformulation by region means that this will not bias our results. In addition, we detect relative reduction in the number of packs purchased as well as energy and nutrients purchased, strengthening the case for consumer behaviour change.

8) Results on food category outcomes. The direction of the differences are unclear. For example, energy & sugar amounts from Sugary drinks, Sugary Cereals & Savory snacks were higher (not significantly) post-policy compared to the counterfactual, but the text does not make this clear and in fact the text.

We have now mentioned the direction of change for non-significant results in our Results section.

9) If the data/analyses already included a control group/sample of North Englanders, why not just do a DID analyses and not bother with a controlled ITS model with the construction of counterfactuals? It is unclear to me if the inclusion of the control sample in the model biases the estimates. Did the authors compare the results from a straightforward DID with covariates vs using a counterfactual?
Several approaches could have been used to analyse these data. Difference in difference has been traditionally more common in health economics and public health policy research (e.g. [https://www.annualreviews.org/doi/abs/10.1146/annurev-publhealth-040617-013507](https://protect-eu.mimecast.com/s/2W4FC8EVLUwDQ0Ku1oJB1?domain=annualreviews.org)) and is generally more frequently reported in medical journals (e.g. [https://www.bmj.com/content/346/bmj.f264.long](https://protect-eu.mimecast.com/s/CJ88C9gWMIYLxXpu3b-j7?domain=bmj.com)), whereas interrupted time series has become popular in (clinical) epidemiology and educational policy (e.g. [https://pubmed.ncbi.nlm.nih.gov/27283160/](https://protect-eu.mimecast.com/s/RVo1C0LKzCrq45LhWgsGx?domain=pubmed.ncbi.nlm.nih.gov)). However, despite these disciplinary differences, the two methods are very similar. In a recent paper, Fry and Hatfield (2021) ([https://onlinelibrary.wiley.com/doi/epdf/10.1111/1475-6773.13697](https://protect-eu.mimecast.com/s/sLWDCg204iYONR7uESDbG?domain=onlinelibrary.wiley.com)) have formally demonstrated the similarities between the two approaches.

In this instance, we chose to use a CITS with a step and trend change to allow the intervention effect to change over time (note that we do not comment on step and trend changes because we use the two-part model where coefficients do not have a straightforward interpretation and hence use the marginal effects). We feel that a CITS design has several important advantages. By modelling the underlying trend, ITS controls for within-group characteristics that tend to change only slowly over time, secular changes, random fluctuations from one time point to the next and regression to the mean. Furthermore, as our evaluation is based on observing a single population (i.e. London households) over time, the CITS design is free from problems due to between-group differences, such as selection bias or unmeasured confounders. We therefore believe that CITS is an appropriate approach for this particular study (<https://academic.oup.com/ije/article/47/6/2082/5049576>).

Given that we observe attenuation of growth in purchases, the use of a control was crucial. Not including a control would have led us to conclude that there had been an increase in the purchases, which would have been biased as an increase was also observed in the control area. We believe that inclusion of the control sample did not bias the estimates in our CITS, as there were parallel pre-intervention trends for all outcomes (see lines 345-352), except for chocolate and confectionery (for which we did an additional sensitivity analysis to test the robustness of this finding and obtained similar results) (see lines 374-375). Using a DiD design would similarly have made use of the control sample.

10) Figure 2 is rather blurry and the quality needs to be improved.
We have provided a higher quality figure.

11) Was the study adequately powered to detect statistical significance for all main outcomes? Some indication of this would be useful.

There is no definitive guidance on power calculations for ITS (<https://protect-eu.mimecast.com/s/YVwzCl714TzKwZESVqClh?domain=ncbi.nlm.nih.gov>) and no prior data to base such calculations on. However, we used the maximum sample we could based on the data Kantar provided. This provided a large number of observations through the number of time points (80 weeks) and households per time point (1970 households). In total, we had 139,193 observations (18,407 observations were dropped due to no reported purchases). Therefore, our study was likely adequately powered to detect differences in the main outcomes. Our findings show that our analysis had sufficient power to detect a 198 kcal (7%) relative change for puddings & biscuits and 0.7 pack (5%) relative change in total HFSS. However, we might have been underpowered for our subgroup analyses, which we mention in the paper (see lines 270-273; 397-406 and 534-540). Better powered studies are needed to look at sub-group differences in advertising policy impact.

12) Given the many outcomes, did the authors consider correcting for this? Bonferroni or Benjamini corrections may be needed.

In total, we tested 36 outcomes, consisting of 6 outcome measures (energy, fat, saturated fat, sugar, salt and packs) for 6 HFSS categories (total, chocolate & confectionery, puddings & biscuits, sugary drinks, sugary cereals and savoury snacks). Our findings do not pass the Bonferroni threshold of p~0.001. We now mention this in the manuscript (lines 445-446). Our findings could be due to chance, however, we detected changes in HFSS purchases at the time of the intervention, and not at another time, in the intervention group relative to the counterfactual. We do not detect changes in non-HFSS purchases. Changes were largest in chocolate and confectionery, which is least likely to have non-HFSS substitutes and continued brand advertising. There was an indication of larger relative reductions among transport users, who were likely to have larger changes in their advertising exposure as a result of the TfL policy. We also found similar or larger changes using a mixed-effects negative binomial model to triangulate our findings. All these sensitivity analyses were used to test the robustness of our findings and point to our findings being true changes rather than chance findings (see lines 446-452).

13) Might the authors say anything about how a longer period under the TfL policy might have a bigger impact given the 10 month post- policy data used?? Would an event study design be possible to address this question?

We were unable to explore changes beyond 10-months post-intervention due to the pandemic and the impact of lockdown on both exposure to outdoor advertising and in purchasing behaviours. However, when we look at the changes each week over the study period, the difference between the observed and counterfactual values gets bigger over time (see Figure 3). We now also compare the changes detected in the first and last post-intervention weeks (see lines 263-266; 362-364 and 526-532 and Supplementary Table S10). We found larger differences in the last post-intervention week compared to the first, which suggests that the changes could be sustained (and even increasing) over time. This analysis is hypothesis generating only and further research is needed to investigate the sustainability of advertising restriction policies.

14) Some version of Supplementary table 10 should be included in the main text as those values are useful to have as reference easily.

We have now moved Supplementary Table S10 to the main text as Table 3.

15) Can the authors say anything about what is known with regards to compliance with the policy? How was it being enforced? What were the penalties?
All advertisements were vetted by TfL for compliance with their policy prior to approving the advertisement. There were no penalties, as non-compliant advertisements would not have been approved to be advertised on the TfL estate. Although there is a possibility that some old, non-compliant advertisements may not have been removed by 25^th^ February 2019, all newly approved advertisements were compliant at time of implementation. Further, there is an exceptions process through which companies can apply to advertise products that are classified as HFSS by the nutrient profiling model, by providing evidence that the product does not contribute to childhood obesity. This process has not been widely used and fewer than 5 products (e.g. stock cubes) were allowed to be advertised. Compliance was therefore high and companies generally did not try to use the exceptions process to circumvent the policy. Companies were able to continue advertising their brand through their non-HFSS products or non-specific brand advertising. We have added these details to the manuscript (see lines 506-524).

16) The analysis does a pre-post and is unable to delve into the mechanisms or monitor actual exposure to unhealthy food marketing (much of which is likely subliminal) and thus is limited to behavioral measures. Please add a short discussion around this and the need for research to better understand the mechanistic /theory of change.

We have now added the need to explore the mechanisms behind the observed changes and change in advertising exposure in the Discussion (see lines 563-565).
