# Supplementary material for: Changes in household food and drink purchases following restrictions on the advertisement of high fat, salt, and sugar products across the Transport for London network: A controlled interrupted time series analysis
Source: PLoS Med. 2022 Feb 17;19(2):e1003915. doi: 10.1371/journal.pmed.1003915 (PMC8853584; doi:10.1371/journal.pmed.1003915)
Supplement: Attachment — Submitted filename: PMEDICINE-D-21-04172R2_ResponsetoReviewers.docx [file pmed.1003915.s025.docx]

We thank the editors and reviewers for their comments on our manuscript. We are glad that our revisions and responses adequately addressed comments from the previous round of review. We have now addressed the remaining comments and responded to them below.

Requests from Editors:

At line 52 (abstract) and elsewhere we suggest avoiding the implied double negative ("-19.4% ... lower", in favour of "19.4% ... lower"; or "-19.4% change in ...").

We have now removed all instances of double negatives from the text.

At line 56, please adapt the language to avoid implying causality: "... so the policy was associated with attenuated growth ... rather than with reduced purchases ...". Please make similar changes throughout the paper where needed.
We have now amended the text to remove any implication of causality.

At line 553, please make that "... possible longer-term impacts".

We have amended this sentence as suggested.

At line 560 and any other instances, please make that "... apparent effect sizes".

We have amended this sentence as suggested.

At line 569, please soften the language used: "... we observed a relative reduction ..." or similar.

We have amended this sentence as suggested.

Noting reference 32, please ensure that all citations have full access details.

We have gone through the reference list to ensure that all citations have full access details.

Comments from Reviewers:

*** Reviewer #1:

Thanks for the revised manuscript and responses to my original queries. Overall, I am happy with the updates and recommend the manuscript be accepted.

One small change - I do not think that incomplete household data is a big problem for this study - but if this was missing not-at-random it could change the findings (i.e. households not reporting data on weeks with a 'treat-heavy' shop). A simple acknowledgement of this as a limitation should be fine.
We have now acknowledged this potential limitation in the Discussion (lines 465-467).

I agree with the authors about ITS vs DID (in the response to reviewers of the first version of the MS) - the advantage of ITS is that it's possible to directly quantify any slope (trend) changes (and overcome several biases) which gives much more information than a simple (adjusted) before-after difference.
Figure 1 helps understand the control locations, and Figure 2 is much easier to read now with adjusted line widths. Sub-group analysis looks fine now.

*** Reviewer #2:

All comments have been adequately addressed

*** Reviewer #3:

From what I can tell, the authors have adequately addressed the comments raised by the reviewers. This is an important paper and it would be good to have this published.
